# Lightweight Anonymous Group Authentication and Quantum-Cloud Key Distribution Based on PUF for Classical Network Environments

**DOI:** 10.3390/s26154840

**Published:** 2026-07-31

**Authors:** Huanjie Zhang, Yang Chen, Shenghao Chen, Zilong Zhao, Dexin Zhu

**Affiliations:** 1 College of Computer Science and Technology, Changchun University, Changchun 130022, China; 241503541@mails.ccu.edu.cn (H.Z.); 241502527@mails.ccu.edu.cn (Y.C.); 251502573@mails.ccu.edu.cn (S.C.); 231501507@mails.ccu.edu.cn (Z.Z.); 2 School of Cyberspace Science and Technology, Beijing Institute of Technology, Beijing 100811, China

**Keywords:** IoT, group key distribution, physical unclonable function (PUF), quantum key cloud server, secure communication

## Abstract

With the rapid development of quantum computing, in response to its disruptive threats to traditional cryptosystems and the urgent demand for lightweight and highly scalable secure group communication among resource-constrained devices in large-scale Internet of Things (IoT) scenarios, this paper proposes a lightweight anonymous group authentication scheme that integrates Physical Unclonable Functions (PUFs), distributed Gossip algorithms, and quantum key distribution. By exploiting the uniqueness and unclonability derived from the inherent physical characteristics of PUF hardware, the scheme fundamentally eliminates attack vectors against quantum computers without requiring devices to pre-store any secret keys in their memory, while the QKCS pre-provisions CRPs and key seeds, which is the standard enrollment procedure in PUF-based systems. Combined with information-theoretically secure quantum keys as session keys, it forms a dual protection mechanism: anti-forgery at the physical layer and anti-quantum attack at the cryptographic layer. Innovatively, the Gossip algorithm is deeply integrated with group key agreement, converting global broadcast into local iterative interactions between nodes, which effectively alleviates broadcast storms and improves the scalability and fault tolerance of the protocol. Meanwhile, a pseudonym mechanism is introduced to achieve anonymous identity protection, and a dynamic key update strategy guarantees forward and backward security when members join or leave the group. Formal verification based on BAN logic and security analysis show that the proposed protocol can resist typical attacks such as replay attacks, man-in-the-middle attacks, and impersonation attacks. Performance evaluations demonstrate that our scheme outperforms existing comparable schemes in terms of computational cost, communication overhead, and dynamic group management efficiency, demonstrating its potential for resource-constrained IoT environments, pending further validation on real hardware platforms.

## 1. Introduction

With the rapid popularization of Internet of Things (IoT) technologies, resource-constrained devices such as sensors and smart terminals have been widely deployed in critical scenarios, including smart grids, the industrial Internet of Things, and the Internet of Vehicles [1]. Most of these applications adopt one-to-many or many-to-many group communication models, in which secure group key distribution and identity authentication are core to ensuring data confidentiality, integrity, and privacy. However, traditional group key management schemes generally rely on computationally hard problems such as integer factorization and discrete logarithms. Their highly complex public-key operations can hardly fit IoT devices with severely limited computing and storage resources. In addition, most existing distributed group protocols use global broadcast mechanisms. As network scale expands, they tend to cause broadcast storms, message loss, and communication delays, resulting in poor robustness and scalability. Some schemes adopt pre-shared keys or digital certificates, which bring risks of key storage leakage and physical attacks, and can hardly achieve anonymous protection for device identities. Therefore, designing post-quantum secure, lightweight, scalable, and anonymous group authentication and key distribution schemes has become a critical issue to be urgently addressed in the field of IoT security [2]. Particularly importantly, many core application scenarios of the IoT, such as wide-area control command delivery in smart grids [3], collaborative control in industrial IoT [4], and vehicle platoon communication in V2X [5], are essentially one-to-many or many-to-many group communication modes. In this mode, a group of devices needs to receive the same control commands or share the same sensing data. Therefore, achieving secure and efficient group communication is the foundation for the implementation of these IoT applications. Given that most IoT devices are only equipped with limited computing resources and energy budgets in their design, they usually have low CPU frequency, memory capacity, and storage capability and need to perform fixed or a small number of tasks during long-term operation [6]. To reduce communication energy consumption and improve efficiency, more and more IoT applications put forward higher requirements for group communication. Realizing secure and efficient group communication requires encrypting messages with a group key to ensure that only legitimate members under the same group key can successfully decrypt and access them. Therefore, the reliable generation and secure distribution of group keys, and member identity authentication, constitute the core technical issues for realizing a secure group communication mechanism.

For IoT security, symmetric cryptography or hash functions are more suitable than asymmetric cryptography because, except for elliptic curve cryptography (ECC) [7,8,9,10,11,12,13,14,15], their computational overhead is much lower than that of asymmetric cryptography. However, the rapid development of quantum computing technology poses a serious threat to these traditional encryption algorithms, necessitating a shift to quantum-safe cryptography. Hybrid encryption systems that combine at least one classical encryption algorithm and one quantum-resistant encryption algorithm are becoming the standard for ensuring communication security. Physical Unclonable Function (PUF), as an integrated circuit (IC), reduces the computational overhead of encryption operations and possesses quantum-resistant properties, with extensive application examples especially in authentication and key distribution [16,17,18,19,20]. In addition to IoT security, the increase in the number of IoT devices may lead to network congestion, message loss, and communication delays. For instance, some existing distributed negotiation schemes [21] rely heavily on global broadcasting and serial computing. As the network scale expands, the probability of broadcast collisions rises sharply, and a single packet loss can easily cause the entire protocol to fail, resulting in poor robustness and scalability. Furthermore, devices in edge computing scenarios such as IoT are usually resource-constrained and cannot withstand cryptography operations with high computational complexity, placing higher requirements on the lightweight design of schemes.

Group communication algorithms are usually applied in IoT-based solutions to execute security primitives at a minimum cost [22]. The main bottleneck of these methods lies in the cost of the key update process rather than group key distribution during node authentication and initialization. Since information is encrypted using the group key, adding new nodes to or expelling existing nodes from the group requires updating the group key to maintain forward and backward secrecy. While updating the group key, this process should be executed at minimum cost on the network and completed as soon as possible. The existing methods are not suitable for IoT devices with extremely limited computing and storage capabilities because they do not consider lightweight encryption primitives, storage, and communication overhead in the same design. Therefore, our design of a PUF-based lightweight group anonymous authentication and key distribution protocol is fully compatible with IoT devices. To eliminate expensive public key cryptography (PKC) operations without compromising security, the low-cost and energy-efficient structure of PUF is the preferred choice.

The Physical Unclonable Function (PUF) achieves uniqueness and unclonability based on physical variations introduced during chip manufacturing. It exhibits attractive merits, such as light weight, physical tamper resistance, no need for pre-stored keys, and inherent quantum resistance, making it highly suitable for resource-constrained devices. Quantum key distribution can provide information-theoretically secure session keys, which further enhances the level of communication security. The distributed Gossip algorithm achieves global state consistency through local iterative interactions among nodes, which can effectively mitigate the broadcast storm problem. However, the existing studies have not effectively integrated the three techniques, making it difficult to simultaneously meet multiple requirements, including lightweight computation, quantum resistance, anonymity, scalability, and dynamic group management. To address the above challenges, this paper proposes a lightweight group authentication and quantum-secure key distribution scheme that integrates the Physical Unclonable Function (PUF), quantum key distribution, and the distributed Gossip algorithm. Based on the physical unclonability of PUF, the scheme presents a lightweight anonymous authentication and group key distribution protocol. Different from traditional cryptography that relies on the complexity of mathematical problems, the quantum resistance of PUF originates from the uniqueness and randomness of hardware physical characteristics, which fundamentally eliminates the attack surface of classical cryptography against quantum computers. The protocol relies only on lightweight operations, including XOR, hash, and PUF evaluations, thereby overcoming the limited computing capability of sensors in the Internet of Things. All keys in the protocol are generated in real time by the PUF, so sensors do not need to store additional keys. This innovation greatly alleviates the storage pressure of sensor devices in IoT scenarios. In addition, we innovatively integrate the Gossip algorithm into the PUF-based key agreement protocol to improve the robustness of the protocol. Compared with the DGAKA protocol proposed by Lee et al. [23], our scheme avoids the problem of broadcast flooding caused by full-group broadcasting, which leads to network congestion and packet loss, and transforms the global group key generation process into a local and iterative convergence process. We enable all nodes to collaborate to iteratively calculate a consistent public value, which is used as the seed for generating the group key. Each node only performs multiple iterative exchanges with its direct neighbors, and finally, all nodes converge to the same value. As a more practical and robust scheme, although it requires multiple rounds of iteration, each round involves reliable and local communication, resulting in a high final success rate and more predictable overall verification time. The scheme uses quantum keys with high randomness as the group key for group communication, which are encrypted and transmitted to each node in the group with the temporary group key, thereby realizing the secure distribution of group quantum keys. The contributions of this paper are as follows:

(1) This paper proposes a PUF-based lightweight anonymous group quantum key distribution protocol, which mainly adopts lightweight operations such as PUF, hash, and XOR instead of computationally expensive modular exponentiation. Leveraging the physical unclonability of PUF, the protocol relies on the uniqueness and randomness of hardware physical characteristics rather than the “mathematical problem complexity” of traditional cryptography, which fundamentally avoids the quantum computer’s cracking path for classical cryptography. Meanwhile, quantum keys are introduced as session keys to enhance information-theoretic security, endowing the proposed protocol with superior efficiency and security compared to other traditional schemes.
(2) In traditional methods, pre-shared keys (PSKs) or digital certificates pose storage leakage risks. In contrast, the proposed scheme utilizes a physically unclonable function (PUF)-generated keys that are dynamically generated in real time based on the physical characteristics of the chip. Member devices are not required to pre-store secret keys, which significantly reduces storage overhead and enhances resistance against physical attacks. Specifically, physical probing will invalidate the response—any attempt at physical access will result in a response completely different from the expected one, as such attempts directly interfere with the PUF result generation process.
(3) Considering the dynamic nature of group communication members, when nodes join or leave the group, our protocol does not require other members to execute all steps of authentication and group key agreement. Compared with other protocols (where all members need to re-perform the entire process), it reduces computational costs and greatly improves key distribution efficiency. Meanwhile, a pseudonym mechanism is introduced—during intra-group authentication and interaction with the quantum key cloud server, pseudonyms instead of real identities are used. This protects member privacy to a certain extent and avoids the direct exposure of identity information.
(4) Aiming at the critical issue of congestion and packet loss caused by global broadcasting in traditional distributed schemes, this study innovatively integrates the Gossip algorithm into the PUF-based key agreement protocol for the first time, transforming the global group key generation into an iterative convergence process of local interactions between nodes. Nodes only exchange and aggregate data with their direct neighbors without the need for full-group broadcasting. Compared with the DGAKA protocol proposed by Lee et al. [23], the proposed scheme significantly reduces the volume of broadcast messages and the probability of network congestion. Furthermore, a single node failure does not affect the global negotiation, leading to substantial improvements in robustness and scalability.
(5) We analyze the security of our proposed scheme using BAN logic and evaluate its performance. Compared with existing IoT key management solutions, the results demonstrate that our scheme is well-suited for resource-constrained IoT environments. By leveraging PUF, we address the challenges of data storage, transmission security, and computational efficiency faced by IoT devices. The proposed protocol offers higher efficiency and security in both computation and storage than previously developed protocols.

The remainder of this paper is organized as follows. Section 2 provides a comprehensive review of existing solutions in the literature. Section 3 introduces the necessary background, after which Section 4 presents our notation, system model, threat model, and security requirements. Section 5 details the proposed scheme. Section 6 conducts both formal and informal security analyses. Section 7 evaluates the performance of our scheme. Section 8 concludes the paper.

## 2. Related Works

In recent years, with the rapid development of the Internet of Things (IoT) [15], the ever-increasing group size has become a tremendous challenge for computationally limited IoT devices and secure group communications. Existing schemes for establishing a group key in IoT environments can be broadly categorized into two classes: group-key agreement protocols and group-key distribution protocols [24,25]. In a group-key agreement protocol, all participants jointly contribute to the construction of the group key without relying on any trusted central authority; the key is determined solely by the members themselves. For example, Naresh et al. [26] proposed a cluster-based hybrid and hierarchical group-key-agreement framework that provides a scalable solution for secure group communications in large wireless ad hoc networks. Xu et al. [27] introduced an anonymous authenticated dynamic group-key-agreement scheme built on blockchain and token mechanisms to improve the efficiency of group communications among IoT devices and to reduce energy consumption in the context of Industry 5.0. Lee et al. [23] presented a lightweight anonymous dynamic group authenticated key-agreement protocol that enables a set of medical sensor devices to mutually authenticate and negotiates a shared session key, addressing secure transmission issues in medical IoT. Wu et al. [28] initiated the notion of asymmetric group key agreement by proposing a single-round asymmetric group-key-agreement (ASGKA) protocol in which a group of users negotiate only one common public encryption key; each user then independently derives his/her own decryption key. The encryption key is publicly available, whereas the decryption keys are kept secret by the individual users. However, this protocol cannot resist active attacks and is only suitable for static groups whose membership remains unchanged. Subsequently, Zhang et al. [29] extended and improved the ASGKA protocol by designing an identity-based authenticated asymmetric group-key-agreement protocol that withstands active attacks and accommodates dynamic membership changes. Zhang Qikun et al. [30] proposed an inter-cluster lightweight asymmetric group-key-agreement protocol for wireless sensor networks to establish secure and efficient group communication channels among sensor nodes in different clusters. Li et al. [31] introduced an attribute-based asymmetric group-key-agreement protocol built on blockchain that enforces access control among protocol participants and addresses secure communication issues in industrial IoT. In distributed group-key-agreement schemes, the trusted center merely distributes secret values to group members during the initialization phase; there is no concept of subgroups, and all members are treated equally.

Group-key distribution is instead determined by a trusted third party called the group-key manager (GKM), which securely delivers the group key to all members [32]; consequently, not every user involved in group communication has to participate in key generation, and users do not need to interact frequently, thereby reducing consumption of time, memory, and processor resources and lowering communication overhead and latency. Numerous schemes have already proposed assorted group-authentication and key-distribution algorithms, mainly built on tree-based structures, Shamir secret sharing, the Chinese Remainder Theorem (CRT), ECC, and other techniques. At present, key distribution falls into two paradigms: centralized and distributed. Distributed key distribution means that a one-time session is collectively decided by all participants; current distributed group-key distribution employs update strategies based on logical trees. Mahalle et al. [33] adopt Paillier threshold cryptography based on Shamir secrets [34] to authenticate and distribute the group key. In this approach, the server generates one public key and multiple private keys for nodes, and then securely distributes the private keys to the nodes. For group operations, every node in the system computes its own message and sends it to the others; all messages are subsequently combined for verification. Only after successful verification are all nodes authenticated; if any node sends an incorrect message, authentication fails. Both methods require cumbersome and complicated operations to achieve authentication and obtain the group key, incurring extremely high overhead. Liu et al. [35] utilize CRT for group-key distribution: authentication and key distribution are executed in two separate phases, and group members can recover the group key via CRT using secrets distributed during registration. Zhou and Ou et al. [36] propose CRT-based group-key management schemes named CRGK and FCRGK. In CRGK, the master node computes the CRT using user information and broadcasts the result; after receiving the broadcast, each user computes the group key with its own key (one modular operation and one XOR). Although the complexity of addition or division for users is constant, the master node must recompute everything from scratch whenever a new group key is required. FCRGK differs from CRGK in that it targets larger groups to accelerate computation during member join/leave events. Another CRT-based secure approach, presented by Kavin and Ganapathy [37], proposes two cryptographic schemes for secure storage and privacy preservation. Shawky et al. [38] suggest implementing group-key distribution via smart contracts on blockchain; however, this method obliges roadside infrastructure to update the smart contract for every vehicle and every group member, which is a notable limitation. Yıldız et al. [39] replace the binary-tree key hierarchy with a factorial tree, conducting an extensive comparison that highlights the factorial tree’s advantages in reducing key-storage cost and the number of key-update messages; tree-based key distribution eliminates the need for a trusted server and addresses key-distribution challenges in widely distributed user scenarios. Cheng et al. [40] propose an anonymous authentication and group-key distribution scheme based on quantum random numbers. In their design, vehicle anonymous certificates are generated by combining random numbers from the vehicle and from the trusted authority, and zero-knowledge proofs are employed by the trusted authority to achieve mutual authentication between vehicles and roadside units. This mechanism guarantees authentication security while effectively protecting vehicle identity privacy. Additionally, the scheme devises a combined key-generation method in which both the roadside unit and the trusted authority jointly contribute to the creation of the group key [41].

## 3. Preliminaries

### 3.1. Physical Unclonable Function

A Physical Unclonable Function (PUF) [42,43,44] refers to an integrated circuit that takes a specific input (referred to as a challenge) and yields a corresponding output (termed a response), with the key characteristic that this response is never retained in digital memory. This relationship can be formally expressed as follows:

$$f:X\to Y,\;X\in {\left\{0,1\right\}}^{\lambda },\;Y\in {\left\{0,1\right\}}^{\eta }$$

In practice, PUF responses are subject to noise caused by temperature [45], voltage, and aging effects. We model the noisy PUF behavior as:

PUF(C)=R+ε,

where *R* is the ideal response, and ε denotes the error vector introduced by environmental variations. To mitigate these errors, we adopt the standard fuzzy extractor approach [46]. During the enrollment phase, helper data is generated from each PUF response and securely stored on the QKCS. During reconstruction, the noisy PUF response is combined with the helper data to reliably recover the same key via error-correcting codes (e.g., BCH or Reed-Solomon codes). The helper data does not reveal the secret key, as it is derived from the difference between the response and a random codeword.

**CRP Management.** To prevent replay and modeling attacks, each challenge-response pair (CRP) is used only once. The QKCS maintains a database of used CRPs and updates it after each protocol execution. When the CRP pool is depleted to a predefined threshold, a secure re-enrollment procedure is triggered to replenish the CRP set. This one-time CRP usage policy ensures that even if an adversary collects a large number of CRPs, the captured data cannot be reused in future sessions.

In essence, a PUF functions as a one-way function where the mapping f converts an input challenge (X) into a unique output response (Y). The uniqueness of this response stems from unavoidable manufacturing discrepancies during integrated circuit (IC) production, which make it practically infeasible to fabricate two chips with identical physical attributes—thus endowing PUFs with inherent unclonability. Additionally, the PUF response is dynamically generated at the exact moment the challenge is applied, rather than being statically stored in memory, preventing direct readout. Any effort to physically probe the PUF architecture will introduce irreversible disturbances, leading to unpredictable alterations in its response and thereby ensuring tamper-resistant security. By leveraging these core properties—uniqueness, unclonability, and tamper resistance—PUFs find extensive applications in authentication, key generation, data integrity protection, access control, and privacy preservation. For security implementation using PUFs, our scheme first pre-selects a set of challenges and stores their corresponding responses prior to the deployment of the quantum-key cloud server; these challenge–response pairs (CRPs) are subsequently utilized to implement security primitives. When the group manager requests a quantum key from the quantum-key cloud server, the server selects one CRP, encrypts the ciphertext using the response $R_{i}$ associated with the challenge $C_{i}$, and then transmits $C_{i}$ to the group manager. Upon receiving $C_{i}$, the group manager inputs it into its local PUF, acquires the identical response $R_{i}$ stored by the cloud server and thereafter employs this response to execute all subsequent operations necessary for decrypting the ciphertext. We note that the invertibility of the keyed BS-PUF described in Section 3.2 does not contradict the above one-way characterization. In the BS-PUF architecture, inversion is only possible with knowledge of the secret key *K*, while without the key, the challenge-response mapping remains computationally irreversible. This is analogous to the semantics of a keyed permutation, such as AES.

### 3.2. Barrel Shifter PUF Architecture of Guo et al. [47]

Guo et al. [47] introduced a novel type of PUF, namely the Barrel Shifter PUF (BS-PUF), wherein a barrel shifter leverages the transmission delay of the circuit to achieve the commutative property and invertibility. They utilized this BS-PUF to develop encryption schemes that possess the commutative and invertible characteristics.

Commutative Property: ${\mathrm{PUF}}_{1}$ and ${\mathrm{PUF}}_{2}$ represent two commutative PUFs. For these PUFs, the outcomes of the composite operations ${\mathrm{PUF}}_{1}\left({\mathrm{PUF}}_{2}\left(X\right)\right)$ and ${\mathrm{PUF}}_{2}\left({\mathrm{PUF}}_{1}\left(X\right)\right)$ must be identical, where *X* denotes an input positive integer.

Invertibility: This attribute relies on an invertible keyed PUF, an input value *X*, and a secret key *K*, which follows the relation:

$$\mathrm{PUF}\left(K,X\right)=Y\Rightarrow {\mathrm{PUF}}^{-1}\left(K,Y\right)=X$$

where ${\mathrm{PUF}}^{-1}$ is derived from the PUF functioning in the reverse direction.

The operational procedures of the BS-PUF are outlined as follows. Initially, a reverse signal is transmitted to the output logic; subsequently, the counter is reset, and a signal is concurrently retransmitted to the output logic. The output logic integrates the signal and delay to accomplish encryption.

The BS-PUF hardware module is physically isolated from the main processor and exposes only two external interfaces: a challenge input port for receiving $C_{i}$, and a response output port for returning $R_{i}$. The PUF response is generated on-the-fly based on physical delay paths and is never stored in any register or memory that can be read by software. Moreover, the internal delay paths and manufacturing variations are not readable through any software command—only the challenge-response evaluation function is accessible. Any physical attempt to probe the PUF circuit permanently alters its delay characteristics, rendering the device unusable for future protocol participation. This hardware-level interface definition justifies that even if a node is semi-trusted or compromised, its software stack cannot export the raw PUF secrets; it can only evaluate the PUF function as intended by the protocol. We also note that BS-PUF has shown resilience against certain machine learning-based modeling attacks. Guo et al. [47] reported that logistic regression attacks failed to model the BS-PUF behavior, which is attributed to the barrel shifter structure that introduces complex, non-linear delay paths that are difficult for linear models to approximate. While this empirical result is encouraging, a comprehensive security evaluation against more advanced modeling attacks (e.g., support vector machines or neural network-based modeling) remains an open direction and is left as future work.

### 3.3. Commutative-PUF Assumption

${\mathrm{PUF}}_{1}$ and ${\mathrm{PUF}}_{2}$ are two commutative ${\mathrm{PUF}}_{s}$. Given two commutative $P_{1}\left(x\right)$ and $P_{2}\left(x\right)$, it is computationally infeasible to compute ${\mathrm{PUF}}_{1}\left({\mathrm{PUF}}_{2}\left(x\right)\right)$ and ${\mathrm{PUF}}_{2}\left({\mathrm{PUF}}_{1}\left(x\right)\right)$ without ${\mathrm{PUF}}_{1}$ and ${\mathrm{PUF}}_{2}$, where *x* is an input positive integer.

### 3.4. Limitation of the Commutative PUF Assumption

The commutative property of PUF used in this paper is based on the Barrel Shifter PUF (BS-PUF) architecture proposed by Guo et al. [47], which provides both commutativity and invertibility through its barrel shifter delay paths. We acknowledge that this assumption may not hold for all PUF implementations. If such a commutative PUF is not available in practice, the proposed protocol can be modified to use a non-commutative PUF with additional key-agreement rounds. This remains a limitation of the current work and a direction for future hardware implementation.

## 4. System Model

### 4.1. Notation Definition

We define the symbols used in this paper: QKCS denotes the quantum-key cloud server, GM denotes the group manager, and U denotes a user; the meanings of other symbols are shown in Table 1.

### 4.2. System Model

**Figure 1:**
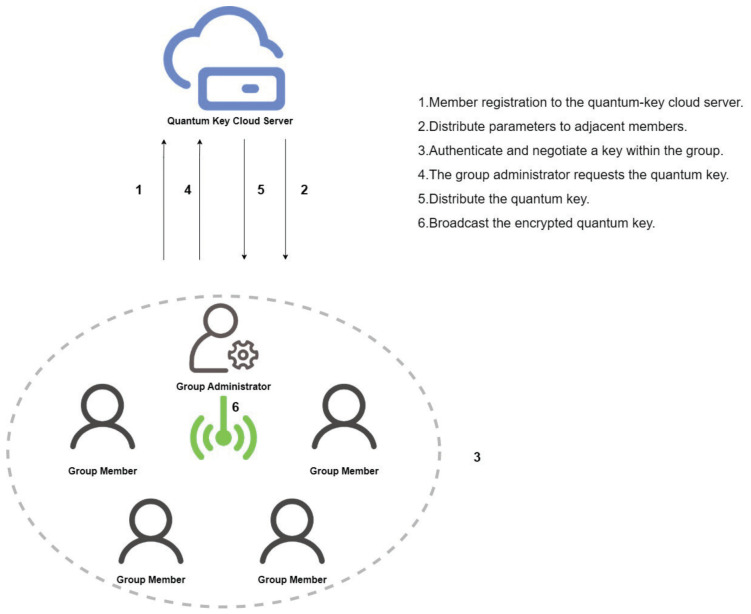
System model diagram.

Referring to the system architecture proposed in [23], the system architecture considered in this study is illustrated in Figure 1. The quantum key cloud server is responsible for distributing quantum keys to the group administrator: it receives quantum key requests from the group administrator and collaborates with the latter to distribute the quantum keys to group members. Group members obtain the quantum keys distributed by the quantum-key cloud server by receiving the ciphertext of the quantum keys broadcast by the group administrator and then decrypting the ciphertext with the group key.

**Quantum-key cloud server:** The quantum-key cloud server stores the identity information of all members. Upon receiving a registration request from a group member, it establishes a shared key with that member. Then, for every two adjacent group members, it transmits a random number protected by their temporary shared key, thereby assisting the members in performing mutual authentication and negotiating a group session key. After the members have successfully agreed on the session key, the server distributes the quantum key QK through an interaction with the group manager.

**Group Manager (GM):** After pairwise mutual authentication among group members and the successful negotiation of the group session key, the GM is responsible for requesting the quantum key from the quantum-key cloud server (QKCS). Upon receiving the request, the QKCS verifies its validity; if the verification succeeds, the server allocates a quantum key QK. To protect QK, the QKCS encrypts it using the response of the CRP challenge–response pair assigned to the GM, XOR-ed with the bitwise sum of all group members’ identity IDs, and transmits the resulting ciphertext *M* to the GM. The GM feeds the challenge $C_{\mathrm{mi}}$ into its own PUF to obtain the response $R_{\mathrm{mi}}$, XORs this response with the same bitwise sum of member IDs, and decrypts *M* to recover QK. Subsequently, the GM encrypts QK with the previously negotiated group key GK and broadcasts the resulting ciphertext to all group members. Each member decrypts the broadcast with GK to obtain QK, and all future intra-group communications are then secured using the quantum key QK.

### 4.3. Threat Model

We adopt the Dolev–Yao (DY) model [47], which involves communication over insecure channels and the distrust between communicating parties. According to this model, a malicious adversary can eavesdrop, modify, delete, forge, replay, or insert fake messages over the insecure public channel between legitimate entities. Naturally, our scheme satisfies the general constraints of secure protocols: even if some session-specific temporary secrets or short-term keys are leaked, the impact on the confidentiality of other long-term keys and communication data in the system should be minimized [48]. Thus, we make the following reasonable assumptions:

**Assumption** **1.** *The quantum-key cloud server (QKCS) is fully trusted. It is equipped with an integrated Quantum Random Number Generator (QRNG), which produces true random numbers based on quantum optical noise; these random numbers serve as the source of the quantum session keys QK. The QKCS honestly executes all procedures, including node registration, identity authentication, pseudonym assignment, PUF challenge-response pair (CRP) management, and quantum key distribution. Its trust boundary encompasses the QRNG hardware module, the key generation software, and the secure CRP database. We explicitly acknowledge that the QKCS constitutes a single point of trust and a high-value target in the system architecture. The server will not actively disclose keys, forge identities, or tamper with the protocol execution. Sensitive data stored by the server, such as node identity information and CRP records, are strictly protected with both physical and logical security guarantees. The QKCS maintains a secure mapping between each node’s real identity, $ID_{i}$, and its current pseudonym, $TID_{i}$, which enables session traceability for regulatory or auditing purposes. The group manager (GM), however, only sees $TID_{i}$ during protocol execution and cannot link different sessions of the same node without access to the QKCS mapping table.*

**Assumption** **2.** *Group managers and ordinary user nodes are semi-trusted. They are regarded as legitimate only at the moment when they pass registration and mutual authentication; we do not guarantee that they remain honest in the long run. A group manager may forge quantum key requests or distribute false information, while an ordinary node may attempt to obtain unauthorized key materials or repeatedly initiate authentication requests. However, all nodes are restricted from exporting the plain text of their own PUF outputs, long-term keys, or digital certificates.*

**Assumption** **3.** *External adversaries can launch typical attacks such as eavesdropping, replay, man-in-the-middle, and impersonation attacks. Internal malicious nodes may try to forge PUF responses, interfere with neighbor interactions, tamper with Gossip iterative messages, or occupy system resources by frequently invoking the protocol, thereby affecting normal execution.*

**Assumption** **4.** *We do not rely on the honesty of any single node, but we trust the unclonability, uniqueness, and physical tamper resistance of Physical Unclonable Functions (PUFs). Any physical probing, cloning, or side-channel attack will directly destroy the hardware characteristics of a PUF and invalidate its responses. An adversary cannot clone or forge the PUF output of a legitimate node in polynomial time. The rationale for trusting that each node can compute its own PUF response while remaining secure is as follows: PUF computation is a physically isolated on-chip process that does not expose any secret material to the software layer. The node’s ability to evaluate ${\mathrm{PUF}}_{i}\left(\cdot \right)$ is a necessary condition for protocol participation, but this ability does not imply that the node can export, clone, or modify the PUF’s physical parameters. In other words, the node is trusted to use the PUF as a black-box function, but the PUF hardware itself is trusted to conceal its internal state from the node’s software and from external adversaries. This distinction between “using” and “reading” the PUF is critical and is now explicitly stated.*

**Assumption** **5.** *We trust the security of the employed cryptographic primitives and components. The one-way hash functions, HMAC, and XOR operations used in this paper satisfy security properties such as collision resistance and pre-image resistance. The Gossip algorithm can achieve global state convergence within a limited number of iterations. Quantum keys have true randomness and information-theoretic security and can resist quantum computing attacks. We emphasize that this information-theoretic security applies to the quantum key itself as a random source; the distribution channel of QK (encrypted under GK and broadcast) remains secured by classical cryptography, not by information-theoretic means.*

### 4.4. Security Objectives

Referring to the security requirements in [31,49], the security objectives that the proposed scheme should satisfy are as follows:

(1) Group Key Security: All communication content during key distribution must possess tamper resistance, and any tampering shall be effectively detected.
(2) Known Key Security: Relay nodes or unauthorized parties cannot obtain key content and plain text information of group communication, so as to protect critical data from leakage.
(3) Perfect Forward Secrecy: Under Assumption 1 of Section 4.3, if the long-term key is compromised at some point in the future, the attacker still cannot decrypt historical communication data. If the QKCS is compromised, forward secrecy is not guaranteed for sessions where the adversary has recorded the full transcript.
(4) Forward Secrecy: When a node leaves the group, it can no longer access the group keys generated thereafter.
(5) Backward Secrecy: When a node joins the group, it cannot decrypt existing historical keys or communication content.
(6) Anonymity: The identities of nodes and the group manager shall remain anonymous during the authentication process to protect their identity privacy and prevent the exposure of sensitive information. Meanwhile, it shall be ensured that all participants are legally registered entities to prevent masqueraded access.
(7) Resistance to Replay Attacks: The scheme design shall prevent attackers from repeatedly sending messages to disrupt the current session.
(8) Resistance to Impersonation Attacks: The scheme design shall prevent unauthorized users from forging communication messages to impersonate legitimate user nodes.
(9) Resistance to Man-in-the-Middle Attacks: The scheme shall prevent attackers from impersonating both communicating parties to intercept messages by forging their identities.

## 5. Proposed Scheme

This section describes the proposed protocol using PUFs for IoMT. It comprises system initialization, member registration phase, authentication, and group key negotiation phases, and quantum key request and distribution phase. The proposed protocol also provides join, leave, fault detection, and tolerance phases for communication within dynamic groups. Assuming that a total of n user have their own independent number, the number range is 1 to n. Assume that user n is a group-manager node with relatively strong computational capability. The details of each phase of the proposed protocols are as follows.

### 5.1. System Initialization

Prior to device deployment, each device is manufactured with an embedded PUF hardware module. No cryptographic keys are pre-stored in the device memory. The quantum-key cloud server stores the identity information of the group manager, selects the corresponding challenge-response pair set $\left(C_{mi},R_{mi}\right)$ for the group manager, locally retains the identity identifiers of all members, and pre-assigns a pre-shared key seed $Qkey_{i}$ to each member. These CRPs and Qkey seeds constitute the only pre-provisioned data. The registration phase itself is conducted over a secure channel before the device is deployed in the field.

The registration message contains $\left(ID_{i},TID_{i},T_{ia},H\left(Qkey_{i}\right),{\mathrm{PUF}}_{a}\left(Qkey_{i}\right)\right)$. Even if an adversary intercepts this message, the following protections apply:

1. $ID_{i}$ is the device’s real identity and is exposed during registration. However, after registration, all subsequent protocol interactions use only the pseudonym $TID_{i}$. The adversary cannot link the intercepted $ID_{i}$ to any later session unless they also possess the pseudonym mapping stored exclusively on the QKCS.
2. $TID_{i}$ is a one-time pseudonym generated as $TID_{i}=H(N_{i}\|ID_{i}\left\|Qkey_{i}\right)$, where $N_{i}$ is a fresh random nonce. The adversary cannot derive $ID_{i}$ or $Qkey_{i}$ from $TID_{i}$ due to the one-way property of the hash function.
3. $H(Qkey_{i})$ is the hash of the pre-shared key seed. The adversary cannot recover $Qkey_{i}$ from its hash due to the one-way property of the hash function.
4. ${\mathrm{PUF}}_{a}\left(Qkey_{i}\right)$ is the PUF response evaluated by the QKCS. Without access to the device’s physical PUF hardware, the adversary cannot reproduce or reuse this response in any meaningful way.

Therefore, interception of a registration message does not compromise device security or enable impersonation in subsequent protocol phases.

### 5.2. Node Registration Phase

1. All group members and the group manager send an application–registration message reqa to the quantum-key cloud server (QKCS) to register themselves; they take the current time-stamp $T_{i}$ and transmit $\left(T_{i},\mathrm{reqa},{\mathrm{ID}}_{i}\right)$ to the QKCS.
2. Upon receiving $\left(T_{i},\mathrm{reqa},{\mathrm{ID}}_{i}\right)$, the quantum-key cloud server first records the current time-stamp $T_{i}^{*}$ and checks the freshness of $T_{i}$ by verifying $\left|T_{i}-T_{i}^{*}\right|<\Delta t.$ After the time-stamp check succeeds, it validates whether the identity ${\mathrm{ID}}_{i}$ exists in its local database. Once the verification is successful, the server selects a random nonce $N_{i}$ and generates a pseudonym for every member as ${\mathrm{TID}}_{i}=H(N_{i}\|{\mathrm{ID}}_{i}\left\|{\mathrm{Qkey}}_{i}\right)$. It then stores the mapping between the real identity ${\mathrm{ID}}_{i}$ and the pseudonym ${\mathrm{TID}}_{i}$. Subsequently, the server feeds the pre-shared key seed ${\mathrm{Qkey}}_{i}$ (allocated to the corresponding user) into its own PUF to compute ${\mathrm{PUF}}_{a}\left({\mathrm{Qkey}}_{i}\right)$, takes the current time-stamp $T_{ai}$, and sends the tuple $\left({\mathrm{ID}}_{i},{\mathrm{TID}}_{i},T_{ai},H\left({\mathrm{Qkey}}_{i}\right),{\mathrm{PUF}}_{a}\left({\mathrm{Qkey}}_{i}\right)\right)$ to each member, including the group manager.
3. Upon receiving the tuple $\left({\mathrm{ID}}_{i},{\mathrm{TID}}_{i},T_{ai},H\left({\mathrm{Qkey}}_{i}\right),{\mathrm{PUF}}_{a}\left({\mathrm{Qkey}}_{i}\right)\right)$, the member first records the current time-stamp $T_{ai}^{*}$ and checks freshness by verifying $\left|T_{ai}-T_{ai}^{*}\right|<\Delta t.$ After the time-stamp check succeeds, the member locally recomputes $H({\mathrm{Qkey}}_{i})$ and compares it with the received value; if the two values match, the member feeds the key seed ${\mathrm{Qkey}}_{i}$ into its own PUF to obtain ${\mathrm{PUF}}_{i}\left({\mathrm{Qkey}}_{i}\right)$, and then derives the shared key with the quantum-key cloud server as $K_{i}={\mathrm{PUF}}_{i}\left({\mathrm{PUF}}_{a}\left({\mathrm{Qkey}}_{i}\right)\right).$ The member then takes the current time-stamp $T_{ia}$ and sends the tuple $\left({\mathrm{ID}}_{i},{\mathrm{TID}}_{i},T_{ia},H\left({\mathrm{Qkey}}_{i}\right),{\mathrm{PUF}}_{i}\left({\mathrm{Qkey}}_{i}\right)\right)$ back to the quantum-key cloud server.
4. Upon receiving the tuple $\left({\mathrm{ID}}_{i},{\mathrm{TID}}_{i},T_{ia},H\left({\mathrm{Qkey}}_{i}\right),{\mathrm{PUF}}_{i}\left({\mathrm{Qkey}}_{i}\right)\right)$, the quantum-key cloud server first records the current time-stamp $T_{ia}^{*}$ and verifies freshness by checking $\left|T_{ia}-T_{ia}^{*}\right|<\Delta t.$ It then compares the received identity–pseudonym pair $\left({\mathrm{ID}}_{i},{\mathrm{TID}}_{i}\right)$ with its local database; if the pair is valid, it recomputes $H({\mathrm{Qkey}}_{i})$ and checks whether it matches the received hash value. Equality implies successful verification. Thereafter, the quantum-key cloud server and the member share the common key
  $$K_{i}={\mathrm{PUF}}_{a}\left({\mathrm{PUF}}_{i}\left({\mathrm{Qkey}}_{i}\right)\right)={\mathrm{PUF}}_{i}\left({\mathrm{PUF}}_{a}\left({\mathrm{Qkey}}_{i}\right)\right).$$

### 5.3. Two-Way Node Authentication and Session Key Negotiation Phase

In this phase, members pairwise negotiate shared keys via PUF(·); each member selects a random nonce $r_{i}$.

**Step 1.** The quantum-key cloud server records the current time-stamp $T_{a}$ and, for every pair $\left(U_{i},U_{i+1}\right)$, chooses a random seed $x_{i}$. It then computes the temporary shared keys

$$TK_{i}=H\;\left({\mathrm{PUF}}_{a}\left({\mathrm{PUF}}_{i}\left({\mathrm{Qkey}}_{i}\right)\right)\left\|T_{a}\right\|{\mathrm{ID}}_{i}\right)$$

$$=H(K_{i}\|T_{a}\|{\mathrm{ID}}_{i})$$

$$TK_{i+1}=H\;\left({\mathrm{PUF}}_{a}\left({\mathrm{PUF}}_{i+1}\left({\mathrm{Qkey}}_{i+1}\right)\right)\left\|T_{a}\right\|{\mathrm{ID}}_{i+1}\right)$$

$$=H(K_{i+1}\|T_{a}\|{\mathrm{ID}}_{i+1})$$

Using $TK_{i}$, it encrypts the random number $x_{i}$ and generates the ciphertext $C_{i}$, together with its MAC:

$$W_{i}=H\left(T_{a}\right)⊕x_{i}$$

$$C_{i}=E_{TK_{i}}({\mathrm{ID}}_{a}\|{\mathrm{ID}}_{i}\|H\left(T_{a}\right)\left\|W_{i}\right)$$

$${\mathrm{MAC}}_{i}={\mathrm{HMAC}}_{TK_{i}}({\mathrm{ID}}_{a}\|{\mathrm{ID}}_{i}\|C_{i}\|H\left(TK_{i}\right)\left\|T_{a}\right)$$

The tuple $\left({\mathrm{ID}}_{q},C_{i},{\mathrm{MAC}}_{i},T_{a}\right)$ is sent to member $U_{i}$. Similarly, using $TK_{i+1}$, the server encrypts the same $x_{i}$ and produces $C_{i+1}$ and ${\mathrm{MAC}}_{i+1}$:

$$W_{i}=H\left(T_{a}\right)⊕x_{i}$$

$$C_{i+1}=E_{TK_{i+1}}({\mathrm{ID}}_{a}\|{\mathrm{ID}}_{i+1}\|H\left(T_{a}\right)\left\|W_{i}\right)$$

$${\mathrm{MAC}}_{i+1}$$

$$={\mathrm{HMAC}}_{TK_{i+1}}({\mathrm{ID}}_{a}\|{\mathrm{ID}}_{i+1}\|C_{i+1}\|H\left(TK_{i+1}\right)\left\|T_{a}\right)$$

and transmits $\left({\mathrm{ID}}_{q},C_{i+1},{\mathrm{MAC}}_{i+1},T_{a}\right)$ to member $U_{i+1}$.

**Step 2.** Upon receiving $\left({\mathrm{ID}}_{q},C_{i},{\mathrm{MAC}}_{i},T_{a}\right)$, member $U_{i}$ first records the current time-stamp $T_{a}^{*}$ and verifies $\left|T_{a}-T_{a}^{*}\right|<\Delta t.$ It then recomputes

$$TK_{i}^{*}=H(K_{i}\|T_{a}\left\|{\mathrm{ID}}_{i}\right)$$

$${\mathrm{MAC}}_{i}^{*}\overset{?}{=}{\mathrm{HMAC}}_{TK_{i}^{*}}({\mathrm{ID}}_{a}\|{\mathrm{ID}}_{i}\|C_{i}\|H\left(TK_{i}^{*}\right)\left\|T_{a}\right)$$

If ${\mathrm{MAC}}_{i}^{*}={\mathrm{MAC}}_{i}$, it decrypts $C_{i}$ and recovers $x_{i}$:

$$D_{TK_{i}^{*}}\left(C_{i}\right)=({\mathrm{ID}}_{a}\|{\mathrm{ID}}_{i}\|H\left(T_{a}\right)\left\|W_{i}\right)$$

$$W_{i}⊕H\left(T_{a}\right)=x_{i}$$

Thereafter, $U_{i}$ computes

$${\mathrm{Tag}}_{i,i+1}^{0}=H\;\left({\mathrm{PUF}}_{i}\left(x_{i}\right)\left\|{\mathrm{TID}}_{i}\right\|H\left(x_{i}\right)\right)$$

$${\mathrm{Tag}}_{i,i+1}^{1}={\mathrm{PUF}}_{i}\left(x_{i}\right)⊕H\left(x_{i}\|{\mathrm{TID}}_{i}\right)$$

and sends $\left({\mathrm{TID}}_{i},{\mathrm{Tag}}_{i,i+1}^{0},{\mathrm{Tag}}_{i,i+1}^{1}\right)$ to $U_{i+1}$.

Analogously, upon receiving $\left({\mathrm{ID}}_{q},C_{i+1},{\mathrm{MAC}}_{i+1},T_{a}\right)$, member $U_{i+1}$ checks the time-stamp, computes

$$TK_{i+1}^{*}=H(K_{i+1}\|T_{a}\left\|{\mathrm{ID}}_{i+1}\right)$$

$${\mathrm{MAC}}_{i+1}^{*}\overset{?}{=}{\mathrm{HMAC}}_{TK_{i+1}^{*}}\left({\mathrm{ID}}_{a}\right\|$$

$${\mathrm{ID}}_{i+1}\|C_{i+1}\|H\left(TK_{i+1}^{*}\right)\left\|T_{a}\right)$$

and, if the MAC is correct, obtains $x_{i}$ via

$$D_{TK_{i+1}^{*}}\left(C_{i+1}\right)=({\mathrm{ID}}_{a}\|{\mathrm{ID}}_{i+1}\|H\left(T_{a}\right)\left\|W_{i}\right)$$

$$W_{i}⊕H\left(T_{a}\right)=x_{i}$$

and finally computes

$${\mathrm{Tag}}_{i+1,i}^{1}={\mathrm{PUF}}_{i+1}\left(x_{i}\right)⊕H\left(x_{i}\|{\mathrm{TID}}_{i+1}\right)$$

**Step 3.** Upon receiving $\left({\mathrm{TID}}_{i},{\mathrm{Tag}}_{i,i+1}^{0},{\mathrm{Tag}}_{i,i+1}^{1}\right)$ from $U_{i}$, member $U_{i+1}$ first computes

$${\mathrm{PUF}}_{i}^{*}\left(x_{i}\right)={\mathrm{Tag}}_{i,i+1}^{1}⊕H\left(x_{i}\|{\mathrm{TID}}_{i}\right)$$

$${\mathrm{Tag}}_{i,i+1}^{0}\overset{?}{=}H\;\left({\mathrm{PUF}}_{i}^{*}\left(x_{i}\right)\left\|{\mathrm{TID}}_{i}\right\|H\left(x_{i}\right)\right)$$

If the equality holds, $U_{i+1}$ chooses a random number, $r_{i+1}$, and records the current time-stamp, $t_{i+1}$. Within one gossip iteration, the following steps are executed:

**Gossip-based aggregation (within one iteration)** [48,50,51] Set the number of rounds,

K=⌈nln100⌉

Compute

$${\mathrm{Tag}}_{i+1,i}^{0}=H\;\left({\mathrm{PUF}}_{i+1}\left(x_{i}\right)\left\|{\mathrm{TID}}_{i+1}\right\|H\left(x_{i}\right)\right)$$

$$K_{i+1,i}={\mathrm{PUF}}_{i+1}\;\left({\mathrm{Tag}}_{i,i+1}^{1}⊕H\left(x_{i}\|{\mathrm{TID}}_{i}\right)\right)$$

$$={\mathrm{PUF}}_{i+1}\left({\mathrm{PUF}}_{i}\left(x_{i}\right)\right)$$

$$SK_{i+1,i}=H\left(K_{i+1,i}\|t_{i+1}\right)$$

Send partial state: set the random number $r_{i+1}$ as the initial value $S_{i+1}^{0}$, and transmit one-half of the current state to the neighbor $U_{i}$:

$$S_{i+1}^{0}=r_{i+1}$$

$$W_{i+1}^{0}=1$$

Update local state: simultaneously receive the value $S_{i+2}^{0}$ from the right neighbor $U_{i+2}$, add the remaining half of its own state, together with all received states, and obtain the new state $S_{i+1}^{1}$ and weight $W_{i+1}^{1}$:

$$S_{i+1}^{1}=\frac{r_{i+1}}{2}+\frac{S_{i+2}^{0}}{2}+\sum \Delta S$$

$$W_{i+1}^{1}=\frac{W_{i+1}^{0}}{2}+\frac{W_{i+2}^{0}}{2}+\sum \Delta W$$

$${\mathrm{Tag}}_{i+1,i}^{2}=SK_{i+1,i}⊕\left(\frac{S_{i+1}^{0}}{2}∥\frac{W_{i+1}^{0}}{2}\right)$$

The current estimate of the global average at node $U_{i+1}$ is $\theta_{i+1}^{1}=\frac{S_{i+1}^{1}}{W_{i+1}^{1}}$ Finally, $U_{i+1}$ sends the tuple $\left({\mathrm{TID}}_{i+1},{\mathrm{Tag}}_{i+1,i}^{0},{\mathrm{Tag}}_{i+1,i}^{1},{\mathrm{Tag}}_{i+1,i}^{2},t_{i+1}\right)$ to $U_{i}$.

**Definition of Quantization Residuals.** In the state update equations, ∑ΔS and ∑ΔW represent the quantization residuals introduced when a node splits its state to transmit to neighbors. Specifically, when the state value cannot be exactly divided by 2 due to finite precision (e.g., when $S_{i}$ is odd in integer arithmetic), the residual $\Delta S=S_{i}-2\cdot \left\lfloor S_{i}/2\right\rfloor$ is introduced to preserve the conservation of the total mass. The same applies to $W_{i}$. This follows the standard quantization handling in Push-Sum gossip implementations as described in [51]. The residuals are accumulated over the averaging process and diminish as the estimates converge.

**Step 4.** Upon receiving the above tuple, $U_{i}$ first records the current time-stamp $t_{i+1}^{*}$ and checks freshness by $\left|t_{i+1}-t_{i+1}^{*}\right|<\Delta t.$ It then computes

$${\mathrm{PUF}}_{i+1}^{*}\left(x_{i}\right)={\mathrm{Tag}}_{i+1,i}^{1}⊕H\left(x_{i}\|{\mathrm{TID}}_{i+1}\right)$$

$${\mathrm{Tag}}_{i+1,i}^{0}\overset{?}{=}H\;\left({\mathrm{PUF}}_{i+1}^{*}\left(x_{i}\right)\left\|{\mathrm{TID}}_{i+1}\right\|H\left(x_{i}\right)\right)$$

$$K_{i,i+1}={\mathrm{PUF}}_{i}\;\left({\mathrm{Tag}}_{i+1,i}^{1}⊕H\left(x_{i}\|{\mathrm{TID}}_{i+1}\right)\right)$$

$$={\mathrm{PUF}}_{i}\left({\mathrm{PUF}}_{i+1}\left(x_{i}\right)\right)$$

$$SK_{i,i+1}=H\left(K_{i,i+1}\|t_{i+1}\right)$$

Now, $U_{i}$ and $U_{i+1}$ share the common key $K_{i,i+1}\;\left(=K_{i+1,i}\right)$ and the temporary session key $SK_{i,i+1}\;\left(=SK_{i+1,i}\right)$ Using $SK_{i,i+1}$, $U_{i}$ decrypts

$$\left(S_{i+1}^{0}/2∥W_{i+1}^{0}/2\right)=SK_{i,i+1}⊕{\mathrm{Tag}}_{i+1,i}^{2}$$

updates its local state

$$S_{i}^{1}=\frac{r_{i}}{2}+\frac{S_{i+1}^{0}}{2}+\sum \Delta S$$

$$W_{i}^{1}=\frac{W_{i}}{2}+\frac{W_{i+1}^{0}}{2}+\sum \Delta W$$

and obtains the current estimate

$$\theta_{i}^{1}=\frac{S_{i}^{1}}{W_{i}^{1}}$$

**Step 5.** After *K* rounds, all nodal estimates converge to the same global average, i.e., $\theta_{i}^{K}$ (i=1,2,…,n) become identical:

$$\theta_{i}^{K}=\theta_{i+1}^{K}$$

To ensure that all nodes derive exactly the same group key GK despite the small numerical deviations in the Gossip convergence, we introduce a deterministic quantization step before the hash operation. Since the Gossip convergence guarantees that $|\theta_{i}^{K}-\theta_{j}^{K}|<10^{-2}$ for any two nodes, *i* and *j*, each node first multiplies its local estimate $\theta_{i}^{K}$ by 100 and applies a floor operation, yielding an identical integer value across all nodes. The group key is then computed as:

$$GK=H(\left\lfloor \theta_{i}^{K}\times 100\right\rfloor )$$

This quantization operation does not affect the security of GK, as the rounding loss is negligible compared to the entropy provided by the initial random values $r_{j}$.

**Gossip Convergence Analysis:** In the Push-Sum gossip protocol adopted in this paper, each node *i* maintains a pair of state variables, $S_{i}^{t}$ and $W_{i}^{t}$, where *t* denotes the round index. Initially, we set $S_{i}^{0}=r_{i}$ and $W_{i}^{0}=1$. At each iteration, *t*, every node transmits half of its current state to a neighbor and performs the updates as defined in Step 3 of Section 5.3. To rigorously justify the convergence rate under our randomized pairing schedule, we express the state evolution in vector form. Let $\theta \left(t\right)={\left(\theta_{1}^{t},\theta_{2}^{t},\ldots ,\theta_{n}^{t}\right)}^{⊤}$, where $\theta_{i}^{t}=S_{i}^{t}/W_{i}^{t}$ is the local estimate of node *i* at round *t*. Let $\bar{r}=\frac{1}{n}\sum_{j=1}^{n}r_{j}$ be the global average of the initial random values, and let $1={\left(1,1,\ldots ,1\right)}^{⊤}$ denote the all-one vector. According to the update rules, the evolution of the state vector follows

θ(t+1)=P(t)θ(t)

where P(t) is a doubly stochastic matrix determined by the random pairing at round *t*. Since each round generates a uniformly random perfect matching, the expected communication graph is the complete graph $K_{n}$. Consequently, the spectral gap of P(t) satisfies

$$\gamma =1-\lambda_{2}=\Theta \left(1/n\right)$$

as established in the standard randomized gossip spectral theory [48,50,51]. Applying the convergence theorem, the mean-square error is bounded by

$$E\left[∥\theta \left(t\right)-\bar{r}{1∥}^{2}\right]\leq {\left(1-\frac{1}{n}\right)}^{t}\cdot {∥\theta \left(0\right)-\bar{r}1∥}^{2}$$

To achieve the required precision $\epsilon =10^{-2}$, we set

$${\left(1-\frac{1}{n}\right)}^{t}\leq 10^{-2}$$

Solving this inequality yields

$$t\geq \frac{\ln100}{\ln(n/(n-1))}$$

Since ln(n/(n−1))≥1/n, we have t≥nln100≈4.6n. This rigorously gives t=O(n) rounds to reach the fixed precision. Therefore, in our protocol, we set the number of rounds as

K=⌈nln100⌉

which guarantees convergence with high probability. The quantization residuals ∑ΔS and ∑ΔW decay exponentially as $O(2^{-t})$ and do not affect this asymptotic bound. Consequently, after *K* rounds, all nodes obtain the same converged value $\theta_{i}^{K}$ (within the prescribed precision), and thus, each node can independently derive the identical group key

$$GK=H(\left\lfloor \theta_{i}^{k}\times 100\right\rfloor )$$

without requiring any further coordination [52].

### 5.4. Robustness Considerations

The above convergence analysis assumes ideal communication conditions. We briefly discuss the robustness of the Gossip-based aggregation under practical imperfections:

**Finite precision:** Due to limited word length on resource-constrained IoT devices, nodes converge to a neighborhood of the true average rather than the exact value. The quantization error is bounded as $∥\theta_{i}^{t}-\bar{r}∥\leq \epsilon +C\cdot \lambda_{2}^{t}$, where $\lambda_{2}=1-\Theta \left(1/n\right)$ is the second-largest eigenvalue of the expected gossip matrix. This does not affect the correctness of the group key derivation, as the resulting $GK=H(\theta_{i}^{K})$ tolerates small deviations due to the collision resistance of the hash function.

**Packet loss and asynchronous execution:** Packet loss is modeled as independent Bernoulli trials with probability *p*. With timeout-based retransmission, convergence is achieved in O(n/(1−p)) rounds, as the randomized pairing-based gossip requires O(n) rounds to propagate information across the entire group. For asynchronous updates, if the time-averaged communication graph is connected, the convergence guarantee still holds, consistent with the analysis in [48,50].

**Node mobility:** For mobile scenarios, the topology changes over time. We assume that the topology variation is slow relative to the Gossip iteration period. As long as the union of communication graphs over any finite time window is connected, the estimates $\theta_{i}\left(t\right)$ converge to the global average $\bar{r}$.

**Malicious perturbation:** Under Byzantine attacks, where, at most, f<n/3 nodes are malicious, the convergence remains within a bounded error, $O(f/n\cdot \max|r_{j}|)$, as the impact of injected false values is limited by the averaging process. The group manager can detect anomalies by comparing received values with expected ranges.

### 5.5. Quantum-Key Request

The group manager initiates a quantum-key request to the quantum-key cloud server (QKCS); after verifying the request, the server selects a quantum key QK and delivers it to the group manager.

**Step 1.** The group manager generates a random nonce, *N*, records the current time-stamp, $T_{m}$, and forms

$${\mathrm{reqa}}_{m}=N\|{\mathrm{ID}}_{1}\|{\mathrm{ID}}_{2}\left\|\;\cdots \;\right\|{\mathrm{ID}}_{n}$$

**Step 2.** It sends the verification request $\left({\mathrm{ID}}_{n},\;T_{m},\;{\mathrm{reqa}}_{m}\right)$

**Step 3.** Upon receiving the request, the QKCS first records the current time-stamp $T_{m}^{*}$ and checks freshness via $\left|T_{m}-T_{m}^{*}\right|<\Delta t.$ After the time-stamp check succeeds, the server proceeds as follows.

**Step 4.** The server queries its database for ${\mathrm{ID}}_{n}$; if found, the group manager is authenticated. The QKCS then selects the CRP challenge–response pair corresponding to node ${\mathrm{ID}}_{n}$, picks the current time-stamp $T_{qm}$, and computes

$$\begin{array}{c} \mathrm{SUM}={\mathrm{ID}}_{1}+{\mathrm{ID}}_{2}+\;\cdots \;+{\mathrm{ID}}_{n}\\ \mathrm{CH}=QK⊕\mathrm{SUM}\\ M=E_{R_{mi}⊕\mathrm{SUM}}\left(\mathrm{CH}\|{\mathrm{ID}}_{n}\right)\\ {\mathrm{MAC}}_{q}={\mathrm{HMAC}}_{R_{mi}}(C_{mi}\|{\mathrm{ID}}_{q}\|T_{q}\left\|N+1\|M\right) \end{array}$$

The server transmits $\left({\mathrm{MAC}}_{q},\;{\mathrm{ID}}_{q},\;T_{q},\;C_{mi},\;M\right)$ to the group manager.

**Step 5.** After receiving the tuple, the group manager first records the current time-stamp $T_{q}^{*}$ and verifies $\left|T_{q}-T_{q}^{*}\right|<\Delta t.$ It then feeds the received $C_{mi}$ into its own PUF to obtain the response $R_{mi}$, increments the nonce by 1, and computes:

$${\mathrm{MAC}}_{q}^{*}\overset{?}{=}{\mathrm{HMAC}}_{R_{mi}}(C_{mi}\|{\mathrm{ID}}_{q}\|T_{q}\left\|N+1\|M\right)$$

If equality holds, the server is authenticated. The manager decrypts *M* and recomputes SUM:

$$\begin{array}{c} \mathrm{SUM}={\mathrm{ID}}_{1}+{\mathrm{ID}}_{2}+\;\cdots \;+{\mathrm{ID}}_{n}\\ \mathrm{CH}\|{\mathrm{ID}}_{n}=D_{R_{mi}⊕\mathrm{SUM}}\left(M\right)\\ QK=\mathrm{CH}⊕\mathrm{SUM} \end{array}$$

Finally, it encrypts QK with the group key GK and broadcasts

$$\mathrm{Bro}=E_{\mathrm{GK}}\left(QK\right)$$

Each user decrypts the broadcast to obtain the group quantum key:

$$D_{\mathrm{GK}}\left(\mathrm{Bro}\right)=QK$$

### 5.6. Group-Key Update

Whenever membership changes (a member joins or leaves), the group key GK must be renegotiated. After the new GK is agreed upon, the group manager sends a fresh quantum-key request to the QKCS, obtains the new quantum key QK, encrypts it with the updated GK, and broadcasts the result to all members again. The steps performed in this section are adapted from the five steps described in Section 5.3.

#### 5.6.1. Member Join

Suppose a new user, $U_{n+1}$, wishes to join the current group $\left(U_{1},U_{2},\ldots ,U_{n}\right)$; a new group is thereby formed, and a fresh group key must be generated. First, $U_{n+1}$ registers with the quantum-key cloud server. Then, the pairs $\left(U_{n},U_{n+1}\right)$ and $\left(U_{n+1},U_{1}\right)$ execute Steps 1–4 of the intra-group authentication and key-agreement phase, after which $U_{n}$ and $U_{n+1}$ share the key

$$K_{n,n+1}={\mathrm{PUF}}_{n+1}\left({\mathrm{PUF}}_{n}\left(x_{n}^{*}\right)\right)$$

$$={\mathrm{PUF}}_{n}\left({\mathrm{PUF}}_{n+1}\left(x_{n}^{*}\right)\right)=K_{n+1,n}$$

and the temporary shared key $SK_{n,n+1}\;\left(=SK_{n+1,n}\right)$ Similarly, $U_{n+1}$ and $U_{1}$ share the key

$$K_{n+1,1}={\mathrm{PUF}}_{1}\left({\mathrm{PUF}}_{n+1}\left(x_{n+1}\right)\right)$$

$$={\mathrm{PUF}}_{n+1}\left({\mathrm{PUF}}_{1}\left(x_{n+1}\right)\right)=K_{1,n+1}$$

and the temporary shared key $SK_{n+1,1}\;\left(=SK_{1,n+1}\right)$

Other members need not execute the full protocol; they only perform Step 3 of the intra-group authentication and key-agreement phase.

**Step 1.** Here, steps similar to Step 3 in Section 5.3 are executed. For users j=1,2,…,n−1, each $U_{j+1}$ chooses its own random number $r_{j+1}^{*}$ and computes

$$SK_{j+1,j}=H\left(K_{j+1,j}\|t_{j+1}\right)$$

$$S_{j+1}^{0}=r_{j+1}^{*},\;W_{j+1}^{0}=1$$

$$Tag_{j+1,j}^{2}=SK_{j+1,j}⊕\left(S_{j+1}^{0}/2∥W_{j+1}^{0}/2\right)$$

and sends the message $\left({\mathrm{TID}}_{j+1},Tag_{j+1,j}^{2},t_{j+1}\right)$ to $U_{j}$, where $t_{j+1}$ denotes the current time-stamp.

**Step 2.** Here, steps similar to Step 4 in Section 5.3 are executed. For users j=1,2,…,n−1, upon receiving $\left({\mathrm{TID}}_{j+1},Tag_{j+1,j}^{2},t_{j+1}\right)$ from $U_{j+1}$, each $U_{j}$ first records the current time-stamp $t_{j+1}^{*}$ and checks freshness via $\left|t_{j}-t_{j+1}^{*}\right|<\Delta t.$ After the check, it computes

$$SK_{j+1,j}=H\left(K_{j+1,j}\|t_{j+1}\right)$$

$$\left(S_{j+1}^{0}/2∥W_{j+1}^{0}/2\right)=SK_{j+1,j}⊕Tag_{j+1,j}^{2}$$

and updates its local state,

$$S_{j}^{1}=\left(r_{j}/2\right)+\left(S_{j+1}^{0}/2\right)+\sum \Delta S$$

$$W_{j}^{1}=\left(W_{j}/2\right)+\left(W_{j+1}^{0}/2\right)+\sum \Delta W$$

**Step 3.** Here, steps similar to Step 5 in Section 5.3 are executed. After *k* rounds, all nodal estimates converge to the uniform global average $\theta_{i}^{k}$, and the new group key is computed as

$${\mathrm{GK}}^{*}=H\left(\left\lfloor \theta_{i}^{K}\times 100\right\rfloor \right)$$

Thereafter, the group manager executes the quantum-key request procedure: it obtains the quantum key QK from the quantum-key cloud server, encrypts QK with the updated group key ${\mathrm{GK}}^{*}$, and broadcasts the ciphertext. Each member decrypts the broadcast with ${\mathrm{GK}}^{*}$ to recover QK, and all subsequent group communications are protected by the quantum key QK.

#### 5.6.2. Member Leave

If user $U_{l}$ leaves the group, the remaining members $\left(U_{1},U_{2},\ldots ,U_{l-1},U_{l+1},\ldots ,U_{n}\right)$ must negotiate a new group key ${\mathrm{GK}}^{\mathrm{new}}$ as follows.

**Step 1.** For the pair $\left(U_{l-1},U_{l+1}\right)$, the quantum-key cloud server chooses a fresh random number, $x_{l-1}^{*}$, and computes the temporary keys

$$TK_{l-1}=H\;\left({\mathrm{PUF}}_{a}\left({\mathrm{PUF}}_{l-1}\left({\mathrm{Qkey}}_{l-1}\right)\right)\left\|T_{s}\right\|{\mathrm{ID}}_{l-1}\right)$$

$$=H(K_{l-1}\|T_{s}\|{\mathrm{ID}}_{l-1})$$

$$TK_{l+1}=H\;\left({\mathrm{PUF}}_{a}\left({\mathrm{PUF}}_{l+1}\left({\mathrm{Qkey}}_{l+1}\right)\right)\left\|T_{s}\right\|{\mathrm{ID}}_{l+1}\right)$$

$$=H(K_{l+1}\|T_{s}\|{\mathrm{ID}}_{l+1})$$

where $T_{s}$ is the current time-stamp. Using $TK_{l-1}$, it encrypts the random number $x_{l-1}^{*}$ and produces ciphertext $C_{l-1}^{*}$ and $MAC_{l-1}^{*}$:

$$W_{l-1}^{*}=H\left(T_{s}\right)⊕x_{l-1}^{*}$$

$$C_{l-1}^{*}=E_{TK_{l-1}}({\mathrm{ID}}_{a}\|{\mathrm{ID}}_{l-1}\|H\left(T_{s}\right)\left\|W_{l-1}^{*}\right)$$

$${\mathrm{MAC}}_{l-1}^{*}=$$

$${\mathrm{HMAC}}_{TK_{l-1}}({\mathrm{ID}}_{s}\|{\mathrm{ID}}_{l-1}\|C_{l-1}^{*}\|H\left(TK_{l-1}\right)\left\|T_{s}\right)$$

and sends $\left({\mathrm{ID}}_{q},\;C_{l-1}^{*},\;{\mathrm{MAC}}_{l-1}^{*},\;T_{s}\right)$ to $U_{l-1}$. Analogously, using $TK_{l+1}$, it encrypts $x_{l+1}^{*}$ and transmits $\left({\mathrm{ID}}_{q},\;C_{l+1}^{*},\;{\mathrm{MAC}}_{l+1}^{*},\;T_{s}\right)$ to $U_{l+1}$ with

$$W_{l+1}^{*}=H\left(T_{s}\right)⊕x_{l+1}^{*}$$

$$C_{l+1}^{*}=E_{TK_{l+1}}({\mathrm{ID}}_{a}\|{\mathrm{ID}}_{l+1}\|H\left(T_{s}\right)\left\|W_{l+1}^{*}\right)$$

$${\mathrm{MAC}}_{l+1}^{*}=$$

$${\mathrm{HMAC}}_{TK_{l+1}}({\mathrm{ID}}_{s}\|{\mathrm{ID}}_{l+1}\|C_{l+1}^{*}\|H\left(TK_{l+1}\right)\left\|T_{s}\right)$$

**Step 2.** $U_{l-1}$ first records the current time-stamp $T_{s}^{*}$ and verifies $\left|T_{s}-T_{s}^{*}\right|<\Delta t.$ After validating ${\mathrm{MAC}}_{l-1}^{*}$, it computes

$$TK_{l-1}^{*}=H\;\left({\mathrm{PUF}}_{l-1}\left({\mathrm{PUF}}_{a}\left({\mathrm{Qkey}}_{l-1}\right)\right)\left\|T_{s}\right\|{\mathrm{ID}}_{l-1}\right)$$

$$=H(K_{l-1}\|T_{s}\|{\mathrm{ID}}_{l-1})$$

and obtains

$$D_{TK_{l-1}^{*}}\left(C_{l-1}^{*}\right)=({\mathrm{ID}}_{a}\|{\mathrm{ID}}_{l-1}\|H\left(T_{s}\right)\left\|W_{l-1}^{*}\right)$$

$$W_{l-1}^{*}⊕H\left(T_{s}\right)=x_{l-1}^{*}$$

It then computes

$${\mathrm{Tag}}_{l-1,l+1}^{0}=H\;\left({\mathrm{PUF}}_{l-1}\left(x_{l-1}^{*}\right)\left\|{\mathrm{TID}}_{l-1}\right\|H\left(x_{l-1}^{*}\right)\right)$$

$${\mathrm{Tag}}_{l-1,l+1}^{1}={\mathrm{PUF}}_{l-1}\left(x_{l-1}^{*}\right)⊕H\left(x_{l-1}^{*}\|{\mathrm{TID}}_{l-1}\right)$$

and sends $\left({\mathrm{TID}}_{l-1},\;{\mathrm{Tag}}_{l-1,l+1}^{0},\;{\mathrm{Tag}}_{l-1,l+1}^{1}\right)$ to $U_{l+1}$.

Similarly, $U_{l+1}$ first checks the time-stamp, validates ${\mathrm{MAC}}_{l+1}^{*}$, computes

$$TK_{l+1}^{*}=H\;\left({\mathrm{PUF}}_{l+1}\left({\mathrm{PUF}}_{a}\left({\mathrm{Qkey}}_{l+1}\right)\right)\left\|T_{s}\right\|{\mathrm{ID}}_{l+1}\right)$$

$$=H(K_{l+1}\|T_{s}\|{\mathrm{ID}}_{l+1})$$

and obtains

$$D_{TK_{l+1}^{*}}\left(C_{l+1}^{*}\right)=({\mathrm{ID}}_{a}\|{\mathrm{ID}}_{l+1}\|H\left(T_{s}\right)\left\|W_{l+1}^{*}\right)$$

$$W_{l+1}^{*}⊕H\left(T_{s}\right)=x_{l+1}^{*}$$

It then computes

$${\mathrm{Tag}}_{l+1,l-1}^{1}={\mathrm{PUF}}_{l+1}\left(x_{l-1}^{*}\right)⊕H\left(x_{l-1}^{*}\|{\mathrm{TID}}_{l+1}\right)$$

**Step 3.** Here, steps similar to Step 3 in Section 5.3 are executed. Upon receiving $\left({\mathrm{TID}}_{l-1},{\mathrm{Tag}}_{l-1,l+1}^{0},{\mathrm{Tag}}_{l-1,l+1}^{1}\right)$ from $U_{l-1}$, $U_{l+1}$ first computes

$${\mathrm{PUF}}_{l-1}^{*}\left(x_{l-1}^{*}\right)={\mathrm{Tag}}_{l-1,l+1}^{1}⊕H\left(x_{l-1}^{*}\|{\mathrm{TID}}_{l-1}\right)$$

and checks

$${\mathrm{Tag}}_{l-1,l+1}^{0}\overset{?}{=}H\;\left({\mathrm{PUF}}_{l-1}^{*}\left(x_{l-1}^{*}\right)\left\|{\mathrm{TID}}_{l-1}\right\|H\left(x_{l-1}^{*}\right)\right)$$

If the equality holds, $U_{l+1}$ chooses a random number, $r_{l+1}^{*}$, records the current time-stamp, $t_{l+1}$, and computes

$${\mathrm{Tag}}_{l+1,l-1}^{0}\overset{?}{=}H\;\left({\mathrm{PUF}}_{l+1}^{*}\left(x_{l-1}^{*}\right)\left\|{\mathrm{TID}}_{l-1}\right\|H\left(x_{l-1}^{*}\right)\right)$$

$$K_{l+1,l-1}={\mathrm{PUF}}_{l+1}\;\left({\mathrm{Tag}}_{l-1,l+1}^{1}⊕H\left(x_{l-1}^{*}\|{\mathrm{TID}}_{l-1}\right)\right)$$

$$={\mathrm{PUF}}_{l+1}\;\left({\mathrm{PUF}}_{l-1}\left(x_{l-1}^{*}\right)\right)$$

$SK_{l+1,l-1}=H\left(K_{l+1,l-1}\|t_{l+1}\right)$ It then sends its partial state to neighbor $U_{l-1}$:

$$S_{l+1}^{0}=r_{l+1}^{*},\;W_{l+1}^{0}=1$$

updates its local state by receiving $S_{l+2}^{0}$ from right neighbor $U_{l+2}$:

$$S_{l+1}^{1}=\left(r_{l+1}^{*}/2\right)+\left(S_{l+2}^{0}/2\right)+\sum \Delta S$$

$$W_{l+1}^{1}=\left(W_{l+1}^{0}/2\right)+\left(W_{l+2}^{0}/2\right)+\sum \Delta W$$

forms

$${\mathrm{Tag}}_{l+1,l-1}^{2}=SK_{l+1,l-1}⊕\left(S_{l+1}^{0}/2∥W_{l+1}^{0}/2\right)$$

and transmits

$$\left({\mathrm{TID}}_{l+1},\;{\mathrm{Tag}}_{l+1,l-1}^{0},\;{\mathrm{Tag}}_{l+1,l-1}^{1},\;{\mathrm{Tag}}_{l+1,l-1}^{2},\;t_{l+1}\right)$$

to $U_{l-1}$, where $t_{l+1}$ is the current time-stamp.

**Step 4.** Here, steps similar to Step 3 in Section 5.3 are executed. Simultaneously, for every user, i=1,2,…,l−2,l+2,…,n, $U_{i+1}$ chooses a random number $r_{i+1}^{*}$ and computes

$$SK_{i+1,i}=H\left(K_{i+1,i}\|t_{i+1}\right)$$

sets

$$S_{i+1}^{0}=r_{i+1}^{*},\;W_{i+1}^{0}=1$$

updates its local state by receiving $S_{i+2}^{0}$ from right neighbor $U_{i+2}$:

$$S_{i+1}^{1}=\left(r_{i+1}^{*}/2\right)+\left(S_{i+2}^{0}/2\right)+\sum \Delta S$$

$$W_{i+1}^{1}=\left(W_{i+1}^{0}/2\right)+\left(W_{i+2}^{0}/2\right)+\sum \Delta W$$

forms

$${\mathrm{Tag}}_{i+1,i}^{2}=SK_{i+1,i}⊕\left(S_{i+1}^{0}/2∥W_{i+1}^{0}/2\right)$$

and sends $\left({\mathrm{TID}}_{i+1},\;{\mathrm{Tag}}_{i+1,i}^{2},\;t_{i+1}\right)$ to $U_{i}$, where $t_{i+1}$ is the current time-stamp.

**Step 5.** Here, steps similar to Step 4 in Section 5.3 are executed. Upon receiving $\left({\mathrm{TID}}_{l+1},{\mathrm{Tag}}_{l+1,l-1}^{0},{\mathrm{Tag}}_{l+1,l-1}^{1},{\mathrm{Tag}}_{l+1,l-1}^{2},t_{l+1}\right)$ from $U_{l+1}$, $U_{l-1}$ first records the current time-stamp $t_{l+1}^{*}$ and verifies $\left|t_{l+1}-t_{l+1}^{*}\right|<\Delta t.$ After the check, it computes

$${\mathrm{PUF}}_{l+1}\left(x_{l-1}^{*}\right)={\mathrm{Tag}}_{l+1,l-1}^{1}⊕H\left(x_{l-1}^{*}\|{\mathrm{TID}}_{l+1}\right)$$

and checks

$${\mathrm{Tag}}_{l+1,l-1}^{0}\overset{?}{=}H\;\left({\mathrm{PUF}}_{l+1}^{*}\left(x_{l-1}^{*}\right)\left\|{\mathrm{TID}}_{l-1}\right\|H\left(x_{l-1}^{*}\right)\right)$$

If the equality holds, it computes

$$K_{l-1,l+1}={\mathrm{PUF}}_{l-1}\;\left({\mathrm{Tag}}_{l+1,l-1}^{1}⊕H\left(x_{l-1}^{*}\|{\mathrm{TID}}_{l+1}\right)\right)$$

$$={\mathrm{PUF}}_{l-1}\;\left({\mathrm{PUF}}_{l+1}\left(x_{l-1}^{*}\right)\right)$$

$$SK_{l-1,l+1}=H\left(K_{l-1,l+1}\|t_{l+1}\right)$$

obtains

$$\left(S_{l+1}^{0}/2∥W_{l+1}^{0}/2\right)=SK_{l+1,l-1}⊕{\mathrm{Tag}}_{l+1,l-1}^{2}$$

updates its local state,

$$S_{l-1}^{1}=\left(r_{l-1}/2\right)+\left(S_{l+1}^{0}/2\right)+\sum \Delta S$$

$$W_{l-1}^{1}=\left(W_{l-1}/2\right)+\left(W_{l+1}^{0}/2\right)+\sum \Delta W$$

and forms the current estimate

$$\theta_{l-1}^{1}=S_{l-1}^{1}/W_{l-1}^{1}$$

**Step 6.** Here, steps similar to Step 4 in Section 5.3 are executed. Simultaneously, for every user, i=1,2,…,l−2,l+1,…,n, upon receiving $\left({\mathrm{TID}}_{i+1},{\mathrm{Tag}}_{i+1,i}^{2},t_{i+1}\right)$ from $U_{i+1}$, $U_{i}$ first records the current time-stamp, $t_{i+1}^{*}$, and checks freshness via $\left|t_{i+1}-t_{i+1}^{*}\right|<\Delta t.$ After the check, it computes

$$\left(S_{i+1}^{0}/2∥W_{i+1}^{0}/2\right)=SK_{i+1,i}⊕{\mathrm{Tag}}_{i+1,i}^{2}$$

updates its local state

$$S_{i}^{1}=\left(r_{i}/2\right)+\left(S_{i+1}^{0}/2\right)+\sum \Delta S$$

$$W_{i}^{1}=\left(W_{i}/2\right)+\left(W_{i+1}^{0}/2\right)+\sum \Delta W$$

and forms the current estimate

$$\theta_{i}^{1}=S_{i}^{1}/W_{i}^{1}$$

**Step 7.** Here, steps similar to Step 5 in Section 5.3 are executed. After *k* rounds, all nodal estimates converge to the uniform global average $\theta_{i}^{k}$. The new group key is computed as

$${\mathrm{GK}}^{\mathrm{new}}=H\left(\left\lfloor \theta_{i}^{K}\times 100\right\rfloor \right)$$

The group manager then executes the quantum-key request procedure: it obtains the quantum key QK from the quantum-key cloud server, encrypts QK with the updated group key ${\mathrm{GK}}^{\mathrm{new}}$, and broadcasts the ciphertext. Each member decrypts the broadcast with ${\mathrm{GK}}^{\mathrm{new}}$ to recover QK, and all subsequent group communications are protected by the quantum key QK.

### 5.7. Node Fault Detection and Fault Resilience Phase

In the identity-authentication and group-key-agreement phase, after receiving the message $\left({\mathrm{TID}}_{i},\;{\mathrm{Tag}}_{i,i+1}^{0},\;{\mathrm{Tag}}_{i,i+1}^{1}\right)$ from user $U_{i}$, user $U_{i+1}$ checks

$${\mathrm{Tag}}_{i,i+1}^{0}\overset{?}{=}H\;\left({\mathrm{PUF}}_{i}^{*}\left(x_{i}\right)\left\|{\mathrm{TID}}_{i}\right\|H\left(x_{i}\right)\right)$$

Likewise, when user $U_{i}$ receives the message from user $U_{i+1}$, it computes and checks

$${\mathrm{Tag}}_{i+1,i}^{0}\overset{?}{=}H\;\left({\mathrm{PUF}}_{i+1}^{*}\left(x_{i}\right)\left\|{\mathrm{TID}}_{i+1}\right\|H\left(x_{i}\right)\right)$$

Should user $U_{i}$ become faulty, user $U_{i+1}$ will detect this in Step 4 through the above equation. The remaining members $\left(U_{1},U_{2},\ldots ,U_{i-1},U_{i+1},\ldots ,U_{n}\right)$ can then filter out user $U_{i}$ and generate a new group key GK by executing the member-leave procedure described in Section 5.6.2. Consequently, the proposed scheme simultaneously provides fault detection and fault-tolerance capabilities. It is worth noting that, if a node fails during the Gossip aggregation rounds rather than during authentication, the mass conservation of the Push-Sum protocol could be temporarily affected. However, the Gossip phase consists of only $K=\lfloor \log_{2}n\rfloor +2$ rounds and completes within a short time window, making such mid-round failures unlikely in practice. Moreover, if a node fails to respond within the expected timeout, it is treated as a faulty node, and the member-leave procedure is triggered to exclude it and re-establish the group key.

## 6. Security Analysis

### 6.1. Formal Security Analysis

In the present section, we adopt the Burrows–Abadi–Needham (BAN) logic [53] to formally validate core properties of the proposed protocol, including identity authentication, key freshness, and key ownership. As a belief-centric protocol verification framework, BAN logic abstracts real message sequences into idealized messages; it leverages the beliefs, perceptions, statements, and authoritative rights of entities to derive reliable assertions, ultimately verifying whether the protocol satisfies predefined security goals. Prior applications of BAN logic have been documented in [40,54]. Table 2 presents the notations used in BAN logic, along with their corresponding interpretations.

#### 6.1.1. BAN Logic Rules

We present only the BAN logic inference rules utilized in our proof as follows:

(1) **Message Meaning Rule (MM):**

$$\frac{P|\equiv P\overset{K}{⟷}Q,\;P◃{\left\{X\right\}}_{K}}{P|\equiv Q|∼X},\;\frac{P|\equiv P\overset{K}{⟺}Q,\;P◃{\left\{X\right\}}_{K}}{P|\equiv Q|∼X}$$

The first form applies to shared keys. If *P* receives a message *X* encrypted with key *K*, and *P* believes that the key *K* is shared with *Q*, then *P* believes *Q* sent the message *X*. The second form applies to shared secret values, following a similar rationale.

(2) **Freshness Rule (FR):**

$$\frac{P|\equiv \#(X)}{P|\equiv \#(X,Y)}$$

If *P* believes that *X* is fresh, then *P* believes that the entire message containing *X* is fresh.

(3) **Nonce verification rule (NV):**

$$\frac{P|\equiv \#(X),\;P|\equiv Q|∼X}{P|\equiv Q|\equiv X}$$

If *P* believes that *Q* said message *X* and believes that *X* is fresh, then *P* believes that *Q* believes *X*.

(4) **Jurisdiction rule (JR):**

$$\frac{P|\equiv Q|\Rightarrow X,\;P|\equiv Q|\equiv X}{P|\equiv X}$$

If *P* believes that *Q* has authority over message *X* and believes that *Q* believes *X*, then *P* believes *X*.

(5) **Composition rule (CR):**

$$\frac{P|\equiv X,\;P|\equiv Y}{P|\equiv (X,Y)}$$

If *P* believes message *X* and *P* believes message *Y*, then *P* believes the composite message (X,Y).

(6) **Session-key rule (SK):**

The sixth rule can be described as

$$\left[P|\equiv \#K,P|\equiv Q|\equiv P\overset{K}{⟷}Q/P|\equiv P\overset{K}{⟷}Q\right]$$

If P believes that K is fresh and Q acknowledges the shared relationship with P, then P believes that K is a shared key with P and Q.

If *P* sees the message (X,Y), then *P* sees the message *X*. Similarly, if *P* sees a message, *X* encrypted by a key, *K*, and *P* believes this key is shared with *Q*, then *P* sees the message *X*.

(7) **Belief conjunction rule (BC):**

$$\frac{P|\equiv Q|\equiv \{X,Y\}}{P|\equiv Q|\equiv X},\;\frac{P|\equiv Q|∼\{X,Y\}}{P|\equiv Q|∼X},\;$$

$$\frac{P|\equiv \{X,Y\}}{P|\equiv X}$$

If *P* believes that *Q* believes or said a composite message, then *P* believes that *Q* believes or said each individual part. Similarly, if *P* believes the entire message, *P* believes each part of that message.

#### 6.1.2. Security Goals


$$Goal1:U_{i}|\equiv QKCS|\equiv U_{i}\overset{K_{i}}{⟷}QKCS$$

$$Goal2:U_{i+1}|\equiv U_{i}|\equiv U_{i+1}\overset{SK_{i,i+1}}{⟷}U_{i}$$

$$Goal3:U_{i}|\equiv U_{j}|\equiv U_{i}\overset{GK}{⟷}U_{j}$$

$$Goal4:GM|\equiv QKCS|\equiv GM\overset{R_{mi}}{⟷}QKCS$$

$$Goal5:U_{i}|\equiv U_{i}\overset{QK}{⟷}U_{j}$$

#### 6.1.3. Known Conditions

Information 1: The quantum-key cloud server receives the user’s registration request.

$$Message1:QKCS◃(T_{i},\mathrm{reqa},{\mathrm{ID}}_{i})$$

Information 2: The user receives the response message sent by the quantum-key cloud server.

$$Message2:U_{i}◃({\mathrm{ID}}_{i},{\mathrm{TID}}_{i},T_{ai},$$

$$H\left({\mathrm{Qkey}}_{i}\right),{\mathrm{PUF}}_{a}\left({\mathrm{Qkey}}_{i}\right))$$

Information 3: The quantum-key cloud server receives the message sent by the user.

$$Message3:QKCS◃({\mathrm{ID}}_{i},{\mathrm{TID}}_{i},T_{ia},$$

$$H\left({\mathrm{Qkey}}_{i}\right),{\mathrm{PUF}}_{i}\left({\mathrm{Qkey}}_{i}\right))$$

Information 4: The user receives the ciphertext encrypted by the quantum-key cloud server with the temporary key.

$$Message4:U_{i}◃\left({\mathrm{ID}}_{q},{\left\{C_{i}\right\}}_{TK_{i}},{\mathrm{MAC}}_{i},T_{a}\right)$$

Information 5: User $U_{i+1}$ receives the authentication tag sent by user $U_{i}$.

$$Message5:U_{i+1}◃\left({\mathrm{TID}}_{i},{\mathrm{Tag}}_{i,i+1}^{0},{\mathrm{Tag}}_{i,i+1}^{1}\right)$$

Information 6: User $U_{i}$ receives the authentication tag and random-number-encrypted message sent by user $U_{i+1}$.

$$Message6:U_{i}◃(TID_{i+1},Tag_{i+1,i}^{0},Tag_{i+1,i}^{1}\;,Tag_{i+1,i}^{2}\;,$$

$${\left\{S_{i+1}^{0},W_{i+1}^{0}\right\}}_{SK_{i+1,i}},t_{i+1})$$

Information 7: The quantum-key cloud server receives the quantum-key request message sent by the group manager.

$$Message7:QKCS◃({\mathrm{ID}}_{n},T_{m},{\mathrm{reqa}}_{m})$$

Information 8: The group manager receives the quantum-key-related information encrypted by the quantum-key cloud server.

$$Message8:GM◃({\mathrm{MAC}}_{q},{\mathrm{ID}}_{q},T_{q},{\left\{C_{mi}\right\}}_{R_{mi}⊕SUM},M)$$

Information 9: The user receives the quantum-key-related information encrypted by the group manager with the group key.

$$Message9:U_{i}◃{\left\{QK\right\}}_{GK}$$

#### 6.1.4. Formal Security Assumptions

$$A1:U_{i}|\equiv U_{i}\overset{K_{i}}{⟷}QKCS$$

User $U_{i}$ believes that it shares the PUF-based key $K_{i}$ with the quantum-key cloud server because $K_{i}$ is generated by further applying the PUF function to the results exchanged between both parties.

$$A2:QKCS|\equiv U_{i}\overset{K_{i}}{⟷}QKCS$$

The quantum-key cloud server believes that it shares the key $K_{i}$ with every user, $U_{i}$.

$$A3:U_{i}|\equiv QKCS|\Rightarrow \left(TID_{i},Qkey_{i},QK\;\right)$$

User $U_{i}$ believes that the quantum-key cloud server has authority over the pseudonym ${\mathrm{TID}}_{i}$, the initial key seed ${\mathrm{Qkey}}_{i}$ and the session quantum key QK.

$$A4:GM|\equiv GM\overset{R_{mi}}{⟷}QKCS$$

The group manager believes that it shares the PUF output $R_{mi}={\mathrm{PUF}}_{\mathrm{GM}}\left(C_{mi}\right)$ with the quantum-key cloud server because the server stores the challenge–response pair $\left(C_{mi},R_{mi}\right)$ of the group manager.

$$A5:U_{i}|\equiv U_{i+1}|\Rightarrow PUF_{i+1}\left(x_{i}\right)$$

User $U_{i}$ believes that user $U_{i+1}$ can generate the PUF output corresponding to $x_{i}$ because a physical unclonable function cannot be forged algorithmically or physically.

$$A6:P|\equiv \#(T_{i},T_{a},T_{m},T_{q}\;)$$

All participants believe that the time-stamps $T_{i},T_{a},T_{m},T_{q}$ of each phase are fresh, preventing replay attacks.

$$A7:P|\equiv \#(x_{i},r_{i})$$

Users believe that the random seed $x_{i}$ allocated by the quantum-key cloud server and the random nonce $r_{i}$ generated by themselves are fresh, ensuring that keys are never reused.

A8:P∣≡H(·)

All participants believe that the hash function satisfies collision resistance.

$$A9:U_{i}|\equiv PUF_{i}\left(x_{i}\right)$$

User $U_{i}$ believes that its own PUF output is unique and cannot be forged either algorithmically or physically.

$$A10:U_{i}|\equiv QK|GK$$

User $U_{i}$ believes that the quantum key QK is stored in the quantum-key cloud server and that QK and GK are physically isolated; GK is used immediately and then discarded, so there is no derivable relation between the two.

#### 6.1.5. Verification

We have verified the proposed scheme and achieved the goals by using the above assumptions and the rules of BAN logic.

**Goal 1:** According to Message 2,

$$U_{i}◃\left(ID_{i},TID_{i},T_{ai},H\left(Qkey_{i}\right),PUF_{a}\left(Qkey_{i}\right)\right)$$

By A1, the PUF-based value $PUF_{a}\left(Qkey_{i}\right)$ serves as an implicit authenticator bound to the shared key $K_{i}$ since only the legitimate QKCS and $U_{i}$ can generate the correct PUF output. Thus, $U_{i}$ obtains:

$$U_{i}|\equiv QKCS|∼\left(ID_{i},TID_{i},T_{ai},H\left(Qkey_{i}\right),PUF_{a}\left(Qkey_{i}\right)\right)$$

By A6 and the FR rule: $U_{i}$ believes that the time-stamp $T_{ai}$ and the fresh random nonce $N_{i}$ (used to generate $TID_{i}$) are fresh. The PUF output $PUF_{a}\left(Qkey_{i}\right)$ is not treated as a nonce; its security relies on the physical unclonability of PUF. By NV and JR rules, Goal 1 follows.

**Goal 2:** According to Message 5,

$$U_{i+1}◃\left(TID_{i},Tag_{i,i+1}^{0},Tag_{i,i+1}^{1}\right)$$

The tag values are computed as:

$$Tag_{i,i+1}^{0}=H(PUF_{i}\left(x_{i}\right)\|TID_{i}\left\|H\left(x_{i}\right)\right)\;$$

$$Tag_{i,i+1}^{1}=PUF_{i}\left(x_{i}\right)⊕H\left(x_{i}\|TID_{i}\right)$$

Since $PUF_{i}\left(x_{i}\right)$ can only be generated by the physical PUF of node $U_{i}$, the successful verification of $Tag_{i,i+1}^{0}$ by $U_{i+1}$ implies that the message originated from $U_{i}$. This is a “PUF-based implicit authentication”, not a MM rule application. We, therefore, derive:

$$U_{i+1}|\equiv U_{i}|\equiv U_{i+1}\overset{SK_{i,i+1}}{⟷}U_{i}$$

By A7 and the NV rule, Goal 2 follows.

**Goal 3:** The group key $GK=H(\theta_{i}^{k})$ is derived from the Gossip convergence value $\theta_{i}^{k}$. The freshness of $\theta_{i}^{k}$ follows from the fresh random nonces $r_{j}$ (for j=1,…,n), which are initialized at the start of each session and mixed through the Push–Sum iterations. Although $\theta_{i}^{k}$ is not a nonce, its value is determined by the fresh $r_{j}$ values that were introduced in Step 3 of Section 5.3. By A7 and the FR rule applied to the initial random values $r_{j}$, each node believes that the resulting $\theta_{i}^{k}$ is associated with a fresh session. Therefore, by the Composition and JR rules, Goal 3 follows.

**Goal 4:** According to Message 7,

$$QKCS◃(ID_{n},T_{m},req_{m})$$

The request message contains $req_{m}=N\|ID_{1}\left\|\ldots \right\|ID_{n}$, where *N* is a fresh random nonce chosen by the GM, and $ID_{n}$ is the GM’s registered identity. The QKCS verifies $ID_{n}$ against its database and checks time-stamp $T_{m}$. Since *N* is fresh, and $ID_{n}$ is pre-registered, the QKCS trusts the authenticity of the request origin. This is a “database-authenticated identity verification”, not a MM rule application. We derive:

$$QKCS|\equiv GM|∼(ID_{n},N,ID_{1},\ldots ,ID_{n})$$

By A6 and the NV rule, Goal 4 follows.

According to Message 8,

$$GM◃(MAC_{q},ID_{q},T_{q},{\left\{C_{mi}\right\}}_{R_{mi}⊕SUM},M)$$

By A4 and the MM rule, we obtain V15:

GM∣≡QKCS∣∼(CH,QK)

By A6, V15 and the NV rule, we achieve Goal 4 V16:

$$GM|\equiv QKCS|\equiv GM\overset{R_{mi}}{⟷}QKCS$$

**Goal 5:** According to Message 9,

$$U_{i}◃{\left\{QK\right\}}_{GK}$$

By Goal 3, $U_{i}$ believes that GK is a valid group key shared among all legitimate group members. The MM rule is applied (here, GK is the shared key, not a principal):

$$U_{i}|\equiv GM|∼QK$$

By the Session-Key Rule and the fact that QK is generated by QRNG with true randomness, Goal 5 follows.

### 6.1.6. Limitations of BAN Logic and Supplementary Security Arguments

While BAN logic provides a rigorous framework for verifying authentication and key agreement properties, it involves inherent limitations. Specifically:

1. BAN logic cannot capture XOR algebra, which is used in several steps of our protocol (e.g., $W_{i}=H\left(T_{a}\right)⊕x_{i}$, $Tag^{1}=PUF_{i}\left(x_{i}\right)⊕H\left(x_{i}\|TID_{i}\right)$).
2. BAN logic does not model physical-layer properties such as PUF unclonability and tamper resistance.
3. BAN logic does not directly establish game-theoretic properties such as key indistinguishability, unlinkability, or insider resistance.

To address these limitations, we supplement the BAN proof with:

1. The informal security lemmas in Section 6.2, which cover anonymity, unlinkability, impersonation resistance, and MitM resistance.
2. The extended formal security arguments in Section 6.3, which define adversary capabilities and security games for key indistinguishability, PFS, anonymity, unlinkability, and insider resistance.

We emphasize that BAN logic remains a standard and widely accepted formalism for authentication protocol verification in PUF-based security literature. Our use of BAN logic is consistent with comparable works such as Lee et al. [23] and Cheng et al. [40].

### 6.2. Informal Security Analysis

In line with the security specifications, this section conducts an informal discussion on the intended security capabilities offered by our proposed scheme. The protocol we put forward provides group key security, mutual authentication, known-key security, perfect forward secrecy, forward and backward security, fault detection and tolerance, as well as resistance against replay and man-in-the-middle attacks.

**Lemma** **1.** *Group-Key Security.*

**Proof.**  Due to the physical unclonability of the PUF, an adversary cannot obtain the function ${\mathrm{PUF}}_{i}\left(\cdot \right)$ of user node $U_{i}$. Therefore, the opponent is unable to compute the key

$$K_{i,i+1}={\mathrm{PUF}}_{i}\left({\mathrm{PUF}}_{i+1}\left(x_{i}\right)\right)$$

nor the temporary key

$$SK_{i,i+1}=H\left(K_{i,i+1}\|t_{i+1}\right)$$

Consequently, no information about $r_{i}$, $W_{i}$, or the group key GK can be extracted from the leaked message

$${\mathrm{Tag}}_{i+1,i}^{2}=SK_{i+1,i}⊕\left(S_{i+1}^{0}/2∥W_{i+1}^{0}/2\right)$$

Hence, the group-key security of the protocol relies on the physical unclonability of the PUF. □

**Lemma** **2.** *Mutual Authentication.*

**Proof.**  In our scheme, for every user pair $\left(U_{i},U_{i+1}\right)$, user $U_{i+1}$ authenticates $U_{i}$ in Step 3 by checking

$${\mathrm{Tag}}_{i,i+1}^{0}\overset{?}{=}H({\mathrm{PUF}}_{i}^{*}\left(x_{i}\right)∥{\mathrm{TID}}_{i}∥H\left(x_{i}\right))$$

while user $U_{i}$ authenticates $U_{i+1}$ in Step 4 by verifying

$${\mathrm{Tag}}_{i+1,i}^{0}\overset{?}{=}H({\mathrm{PUF}}_{i+1}^{*}\left(x_{i}\right)∥{\mathrm{TID}}_{i+1}∥H\left(x_{i}\right))$$

Thus, our scheme provides mutual authentication. □

**Lemma** **3.** *Known-Key Security.*

**Proof.**  Since the final group key is

$$\mathrm{GK}=H(\theta_{i}^{k})$$

and θ depends on *r*, where $r_{i}$ is chosen randomly by the user and differs in every session, the group keys GK in distinct sessions are independent. Therefore, our scheme satisfies known-key security. □

**Lemma** **4.** *Perfect Forward Secrecy.*

The proposed scheme provides perfect forward secrecy under the assumption that the QKCS remains uncompromised.

**Proof.**  We first identify the long-term secrets that could be compromised in a realistic attack scenario: (i) PUF responses stored as CRPs on the QKCS; (ii) Qkey seeds; (iii) group manager credentials; and (iv) protocol transcripts. □

We analyze PFS under two distinct scenarios:

Scenario A (QKCS remains trusted): This scenario is consistent with Assumption 1 in Section 4.3. Even if long-term PUF responses, CRPs, Qkey seeds, or GM credentials are compromised, past GK and QK values remain secure. Each GK is derived from ephemeral Gossip convergence values using one-time random nonces $r_{i}$, and each QK is QRNG-generated per session. Recomputing past Gossip iterations is computationally infeasible. Thus, past session keys are protected.

Scenario B (QKCS is compromised): This scenario falls outside Assumption 1 of Section 4.3. If the QKCS is breached, the adversary gains access to all stored CRPs and Qkey seeds. If session transcripts are also available, they can reconstruct past temporary keys, $TK_{i}=H(K_{i}\|T_{a}\left\|ID_{i}\right)$, and decrypt past communications. Therefore, strong PFS cannot be guaranteed if the QKCS is compromised.

We explicitly state this limitation: the proposed scheme provides PFS under Assumption 1 of Section 4.3. If the QKCS is breached, forward secrecy is not guaranteed for sessions where the adversary has recorded the full transcript.

**Lemma** **5.** *Forward and Backward Security.*

**Proof.**  Our scheme supports dynamic group communications with dedicated join and leave phases. When a member joins or leaves, a new group key is generated, and a fresh quantum key is re-requested from the QKCS and broadcast to all current members. Importantly, the first three steps of identity authentication and key agreement do not need to be re-executed upon each membership change. Therefore, join and leave operations do not compromise the security of past or future group keys, satisfying both forward and backward security. □

**Lemma** **6.** *Fault Detection and Fault Tolerance.*

**Proof.**  Our scheme includes a fault-detection and fault-tolerance phase in which faulty node $U_{i}$ can be detected. Other nodes filter out the faulty node $U_{i}$ and execute the member-leave phase to generate a new group key GK, establishing a new secure communication channel; the group manager then distributes a new quantum key. Therefore, the proposed scheme provides fault detection and fault tolerance. □

**Lemma** **7.** *Anonymity and User-Privacy Protection.*

**Proof.**  In all subsequent protocol interactions after registration, the pseudonym ${\mathrm{TID}}_{i}=H(N_{i}∥{\mathrm{ID}}_{i}∥{\mathrm{Qkey}}_{i})$ replaces the real identity ${\mathrm{ID}}_{i}$. The one-way property of H(·) prevents any adversary from recovering ${\mathrm{ID}}_{i}$ from ${\mathrm{TID}}_{i}$. Although ${\mathrm{ID}}_{i}$ is exposed during registration (over a secure channel) and in the quantum key request (by the trusted GM to the trusted QKCS), these exposures do not compromise the protocol’s anonymity, as all subsequent authentication, key agreement, and key distribution steps use only ${\mathrm{TID}}_{i}$. □

**Lemma** **8.** *Resistance to Replay Attacks.*

**Proof.**  Time-stamps $t_{i}$ are used in every message to guarantee freshness; therefore, our scheme can resist replay attacks. □

**Lemma** **9.** *Resistance to Impersonation Attacks.*

**Proof.**  Suppose an adversary, $U_{i}^{*}$, attempts to impersonate node $U_{i}$. It cannot correctly compute

$$TK_{i}=H({\mathrm{PUF}}_{a}\left({\mathrm{PUF}}_{i}\left({\mathrm{Qkey}}_{i}\right)\right)∥T_{a}∥{\mathrm{ID}}_{i})=$$

$$H(K_{i}∥T_{a}∥{\mathrm{ID}}_{i})$$

$$K_{i,i+1}={\mathrm{PUF}}_{i}\left({\mathrm{Tag}}_{i+1,i}^{1}⊕H\left(x_{i}∥{\mathrm{TID}}_{i+1}\right)\right)$$

$$={\mathrm{PUF}}_{i}\left({\mathrm{PUF}}_{i+1}\left(x_{i}\right)\right)$$

$$SK_{i,i+1}=H\left(K_{i,i+1}∥t_{i+1}\right)$$

Due to the physical unclonability of the PUF, no attacker can forge ${\mathrm{PUF}}_{i}$; hence, $U_{i}^{*}$ cannot compute

$${\mathrm{Tag}}_{i,i+1}^{0}=H({\mathrm{PUF}}_{i}\left(x_{i}\right)∥{\mathrm{TID}}_{i}∥H\left(x_{i}\right))$$

$${\mathrm{Tag}}_{i,i+1}^{1}={\mathrm{PUF}}_{i}\left(x_{i}\right)⊕H\left(x_{i}∥{\mathrm{TID}}_{i}\right)$$

and cannot send the correct message $\left({\mathrm{TID}}_{i},{\mathrm{Tag}}_{i,i+1}^{0},{\mathrm{Tag}}_{i,i+1}^{1}\right)$. Therefore, the adversary $U_{i}^{*}$ will be detected in Step 3 via ${\mathrm{Tag}}_{i,i+1}^{0}$, and our scheme resists impersonation attacks. □

**Lemma** **10.** *Resistance to Man-in-the-Middle Attacks.*

**Proof.**  Our scheme realizes mutual authentication between every pair $\left(U_{i},U_{i+1}\right)$ through PUF-based verification: $U_{i+1}$ authenticates $U_{i}$ by verifying ${\mathrm{Tag}}_{i,i+1}^{0}$, and $U_{i}$ authenticates $U_{i+1}$ by verifying ${\mathrm{Tag}}_{i+1,i}^{0}$ (see Lemmas 2 and 9). This ensures that both parties can verify each other’s identity before any sensitive key material is exchanged. Additionally, time-stamps $t_{i}$ are used in every message to guarantee freshness and prevent replay attacks. Consequently, an attacker cannot relay, alter, or replay communications between $U_{i}$ and $U_{i+1}$ without being detected, so both parties believe they are communicating directly. Therefore, our scheme can resist man-in-the-middle attacks. □

### 6.3. Extended Formal Security Arguments and RoR-Based Proof

In this section, we first define the adversary model and security games for the proposed protocol and then provide a formal game-based security proof under the Real-or-Random (RoR) model [55]. Following the methodologies in [56,57], we construct a series of interactive games between a challenger, C, and an adversary, A, to simulate their interactions. Through this process, we rigorously evaluate the ability of A to distinguish the real group key from a random key, thereby defining its formal security advantage.

#### 6.3.1. Adversary Model

We adopt the Dolev–Yao model with the following queries available to the adversary A:

- Execute(P): passive eavesdropping of a complete protocol session;
- Send(P,m): active injection of messages into the protocol;
- Reveal(P): revealing the session key of a completed session;
- Corrupt(P): revealing all long-term secrets of a node;
- Test(P): the challenger returns either the real session key or a random value, and A must distinguish which.

A session is considered “fresh” if its session key has not been revealed and the participating nodes have not been corrupted. The session identifier SID is defined as the tuple of all exchanged messages in the session.

#### 6.3.2. Security Games

The security goals are defined as the following games:

1. **Key Indistinguishability (Game 1).** A issues a Test query on a fresh session. The adversary’s advantage is ${\mathrm{Adv}}_{A}=\left|\mathrm{Pr}\left[\mathrm{success}\right]-1/2\right|$. Under the assumptions that PUF is physically unclonable, the hash function is collision-resistant, and QRNG produces true randomness, ${\mathrm{Adv}}_{A}$ is negligible.
2. **Perfect Forward Secrecy (Game 2).** A is allowed to issue Corrupt queries after session completion. Past session keys remain secure because each QK is one-time and each GK is ephemeral, derived from independent Gossip convergence values. This holds under the assumption that the QKCS remains uncompromised.
3. **Anonymity and Unlinkability (Game 3).** A attempts to link two sessions to the same user. The adversary’s advantage is negligible because a new pseudonym $TID_{i}$ is generated for each session using a fresh random nonce.
4. **Insider Resistance (Game 4).** A corrupts an internal node and attempts to impersonate another node. Due to the physical uniqueness of each node’s PUF, the adversary cannot reproduce the PUF response of the target node.

#### 6.3.3. Formal Security Argument Based on the Real-or-Random Model

We now provide a formal security argument under the defined adversary model and cryptographic assumptions.

The validity of this security argument rests on the following cryptographic and physical assumptions:

(i) The hash function satisfies collision resistance;
(ii) PUF outputs are physically unclonable and unpredictable;
(iii) The BS-PUF architecture satisfies the commutativity property;
(iv) The Gossip convergence values are derived from fresh one-time random nonces and are thus independent across sessions.

Let A be a polynomial-time adversary attempting to compromise the semantic security of the proposed protocol Σ. Its advantage ϵ satisfies:

$$\epsilon \leq q_{h}\cdot {\mathrm{Adv}}_{H}^{\mathrm{Coll}}\left(B_{1}\right)+q_{p}\cdot {\mathrm{Adv}}_{\mathrm{PUF}}^{\mathrm{Unpred}}\left(B_{2}\right)+\left(q_{e}+q_{g}\right)\cdot {\mathrm{Adv}}_{\mathrm{PUF}}^{\mathrm{Comm}}\left(B_{3}\right),$$

where $q_{h}$ denotes the maximum number of hash queries, $q_{p}$ the maximum number of PUF evaluation queries, $q_{e}$ the maximum number of session key establishment queries, and $q_{g}$ the maximum number of Gossip-related queries.

We prove the negligible advantage of the adversary through a sequence of games $G_{0}$ to $G_{5}$. Let $S_{i}$ denote the event that A correctly guesses the random bit *b* in game $G_{i}$.

**Game $G_{0}$:** This is the real protocol execution under the random oracle model. The adversary’s advantage equals its probability of breaking the real protocol:

$$\epsilon =|\mathrm{Pr}[S_{0}]-1/2|.$$

**Game $G_{1}$:** All hash queries are replaced with a random oracle. If the adversary can distinguish $G_{1}$ from $G_{0}$, an algorithm, $B_{1}$, can break the collision resistance of the hash function:

$$|\mathrm{Pr}\left[S_{1}\right]-\mathrm{Pr}\left[S_{0}\right]|\leq q_{h}\cdot {\mathrm{Adv}}_{H}^{\mathrm{Coll}}\left(B_{1}\right).$$

**Game $G_{2}$:** All PUF outputs used in key generation are replaced with random values. If the adversary can distinguish $G_{2}$ from $G_{1}$, an algorithm, $B_{2}$, can break the unpredictability of PUF:

$$|\mathrm{Pr}\left[S_{2}\right]-\mathrm{Pr}\left[S_{1}\right]|\leq q_{p}\cdot {\mathrm{Adv}}_{\mathrm{PUF}}^{\mathrm{Unpred}}\left(B_{2}\right).$$

**Game $G_{3}$:** The shared key $K_{i}$ between each node and the QKCS is replaced with a random value. Since $K_{i}={\mathrm{PUF}}_{a}\left({\mathrm{PUF}}_{i}\left(Qkey_{i}\right)\right)$ relies on the commutativity of PUF, distinguishing $G_{3}$ from $G_{2}$ implies breaking the commutative PUF assumption:

$$|\mathrm{Pr}\left[S_{3}\right]-\mathrm{Pr}\left[S_{2}\right]|\leq q_{e}\cdot {\mathrm{Adv}}_{\mathrm{PUF}}^{\mathrm{Comm}}\left(B_{3}\right).$$

**Game $G_{4}$:** The Gossip convergence value $\theta_{i}^{K}$ is replaced with a random value. Since $\theta_{i}^{K}$ depends on the fresh random nonces $r_{j}$ and the Gossip mixing process, the adversary cannot distinguish it from random without knowing all $r_{j}$:

$$|\mathrm{Pr}\left[S_{4}\right]-\mathrm{Pr}\left[S_{3}\right]|\leq q_{g}\cdot {\mathrm{Adv}}_{\mathrm{Gossip}}^{\mathrm{Dist}}\left(B_{4}\right).$$

**Game $G_{5}$:** The final group key $GK=H(\theta_{i}^{K})$ and the quantum key QK are replaced with random values. Since QK is generated by QRNG with true randomness, the adversary has no advantage:

$$\mathrm{Pr}[S_{5}]=1/2.$$

Combining all games:

$$\begin{array}{cc} \epsilon & =|\mathrm{Pr}\left[S_{0}\right]-1/2|=|\mathrm{Pr}\left[S_{0}\right]-\mathrm{Pr}\left[S_{5}\right]|\\ & \leq \sum_{i=0}^{4}\left|\mathrm{Pr}\left[S_{i}\right]-\mathrm{Pr}\left[S_{i+1}\right]\right|\\ & \leq q_{h}\cdot {\mathrm{Adv}}_{H}^{\mathrm{Coll}}\left(B_{1}\right)+q_{p}\cdot {\mathrm{Adv}}_{\mathrm{PUF}}^{\mathrm{Unpred}}\left(B_{2}\right)+\left(q_{e}+q_{g}\right)\cdot {\mathrm{Adv}}_{\mathrm{PUF}}^{\mathrm{Comm}}\left(B_{3}\right). \end{array}$$

Since all terms are negligible, the adversary’s advantage ϵ is negligible. Therefore, the proposed scheme achieves semantic security under the RoR model. We emphasize that this RoR-based security argument demonstrates the indistinguishability of the group key under the above assumptions. It does not claim to capture all implementation-level attacks (e.g., side-channel attacks) or all practical deployment scenarios.

### 6.4. Compromise Analysis

Compromise of GK: If the current group key GK is exposed, the adversary can decrypt the current broadcast message $Bro=E_{GK}\left(QK\right)$ and obtain the current quantum key QK. However, past QK values remain secure because each QK is used for only one session, and all session keys are independently generated by the QRNG. Forward secrecy for past sessions is thus preserved.

Compromise of GM: If the group manager is compromised, the adversary may obtain the current QK and impersonate the GM for future quantum key requests. However, the QKCS detects anomalies via MAC verification and time-stamp freshness checks on each request. Moreover, without the GM’s physical PUF hardware, the adversary cannot forge the PUF-based authentication messages required in the key request phase.

Compromise of QKCS: This is the most severe scenario. If the QKCS is breached, all stored CRPs, Qkey seeds, and pseudonym mappings are exposed. In this case, strong perfect forward secrecy cannot be guaranteed, because the adversary can reconstruct past temporary keys if session transcripts are also available. We, therefore, explicitly state that perfect forward secrecy holds only under the assumption that the QKCS remains uncompromised.

## 7. Performance Analysis

In this section, we evaluate the performance of our scheme in terms of storage and computational overhead. All experiments were conducted in Python 3.10; the hmac library was invoked for HMAC operations, while SHA-256 hashing was realized through the hashlib module. The test platform is equipped with an Intel(R) Core(TM) i7-10510U CPU @ 1.80 GHz (2.30 GHz), 16 GB RAM, and Windows 10 22H2 (64-bit). We denote by $T_{\mathrm{puf}}$, $T_{h}$, $T_{c}$, $T_{\mathrm{pm}}$, $T_{s}$, $T_{e}$, $T_{\mathrm{hmac}}$ and $T_{\mathrm{sr}}$ the execution times for PUF evaluation, hash function, Chebyshev chaotic map, elliptic-curve point multiplication, symmetric encryption/decryption, modular exponentiation, HMAC computation and Shamir-secret-sharing reconstruction, respectively. Each primitive was executed 1000 times on our test-bed; the average results are listed in Table 3.

### 7.1. Computational Cost Analysis

**Figure 2:**
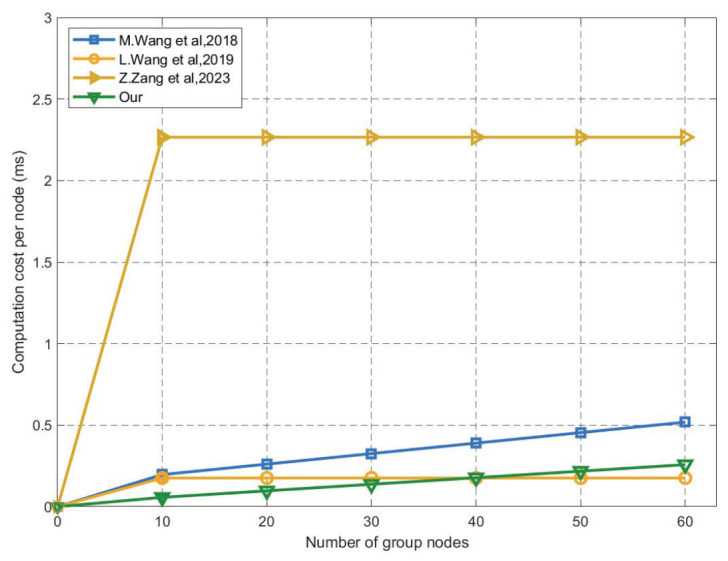
Computation cost comparison of each group node based on the data from Refs. [56,58,59].

**Figure 3:**
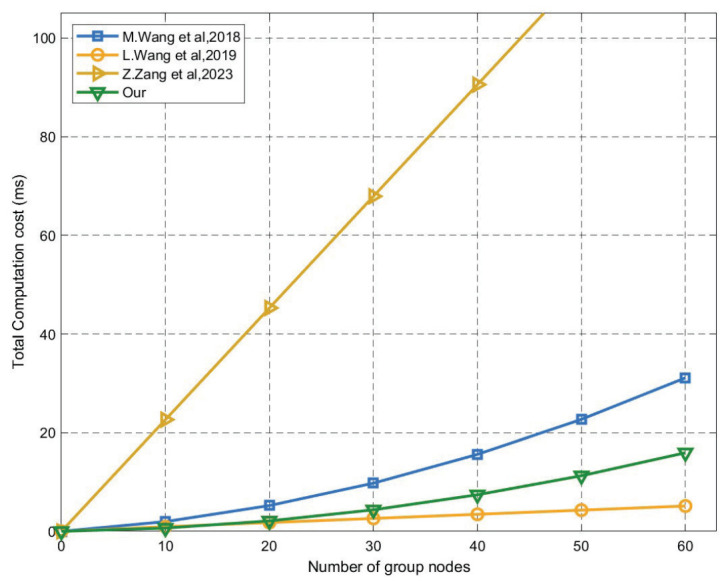
Computation cost comparison of all group nodes based on the data from Refs. [56,58,59].

In this part, we evaluate the computational cost of our protocol and compare it with the overhead of [56,58,59]. We acknowledge that the experimental evaluation was conducted on a desktop-class platform, which does not fully reflect the computational constraints of resource-constrained IoT devices. Nevertheless, we emphasize that the primary contribution of this paper is the protocol design and its comparative performance advantage, rather than hardware-specific optimization. The desktop platform provides a controlled and reproducible environment for fair comparison with existing schemes. A full hardware implementation on real embedded platforms, including energy consumption and storage overhead measurements, is planned as future work. We note that the comparison in Table 4 is made against selected representative lightweight group-key schemes from the recent literature that provide complete complexity formulas for quantitative comparison. This does not constitute an exhaustive comparison with all existing PUF-based, QRNG-assisted, or Gossip-based approaches, as many such works lack fully specified complexity models. To facilitate a concise comparison, we define several cryptographic primitives as shown in Table 3 and measure their execution time under the same unit-cost model. In our protocol, the system-initialization and registration procedures are executed only once at start-up; subsequent member authentication and group-key agreement do not repeat these steps. After initialization, both members and the quantum-key cloud server already share pairwise keys, so—like the cited works—we focus on the pairwise-authentication, group-key-agreement and quantum-key-distribution phases. During authentication and group-key agreement each user node performs 2 PUF evaluations, 7+nln100 hash operations, 1 decryption and 1 HMAC operation. In the quantum-key-reception phase, each user executes 1 decryption. Thus, the per-node computational cost is $2T_{\mathrm{puf}}+\left(n\ln100+7\right)T_{h}+2T_{s}+T_{\mathrm{hmac}}.$ The quantum-key cloud server performs 3 hash operations, 1 encryption and 1 HMAC operation. When the group manager requests the quantum key, the server executes 1 PUF evaluation, 1 HMAC and 1 decryption, while the cloud server itself carries out 1 HMAC and 1 encryption; the group manager needs 1 encryption for broadcasting the quantum key. Hence, the total group-wide cost is $\left(2n+1\right)T_{\mathrm{puf}}+\left(n^{2}\ln100+9n\right)T_{h}+\left(3n+3\right)T_{s}+\left(2n+2\right)T_{\mathrm{hmac}}$ The analysis of the remaining schemes is similar and omitted here. The aggregated results are listed in Table 4. To provide a rough reference for resource-constrained embedded platforms, we estimate that considering a typical 10-30x performance gap between desktop-class systems and ARM Cortex-M class devices reported in the literature, the per-node computational overhead of our protocol would remain within the typical duty cycle of IoT sensors. For example, on an ARM Cortex-M4 class device, the estimated per-node overhead is roughly 0.2–0.6 ms, which is still negligible compared to typical sensor sleep intervals. We emphasize that these are rough estimates based on literature-reported scaling factors; actual embedded-device latency, energy consumption, and memory usage have not yet been experimentally validated and are left as future work. Ref. [58] relies mainly on elliptic-curve point multiplication and modular exponentiation, so its overhead is high. Ref. [59] employs modular exponentiation plus elliptic-curve point multiplication. Ref. [56] adopts the Chebyshev chaotic map, whose cost is much higher than our PUF evaluation. Figure 2 compares the per-user computational cost when the number of nodes increases from 0 to 60, while Figure 3 illustrates the trend of the total group computational cost over the same range. Because our protocol avoids expensive modular exponentiations and Chebyshev-map operations, its advantage becomes more pronounced as the group size grows.

**Figure 4:**
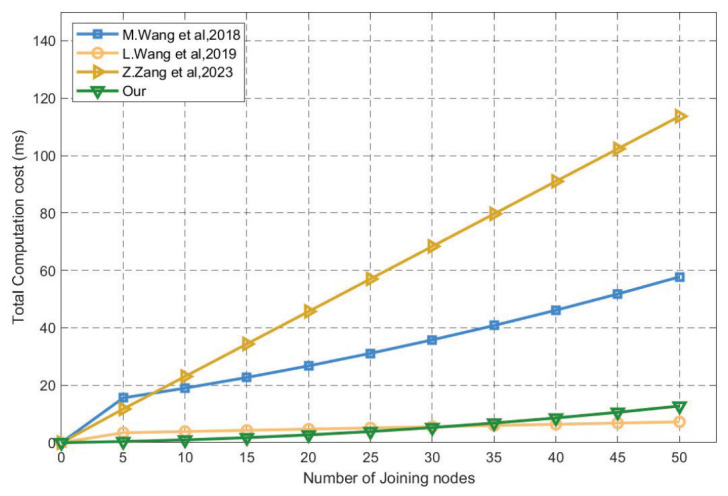
Node joining cost comparison based on the data from Refs. [56,58,59].

**Figure 5:**
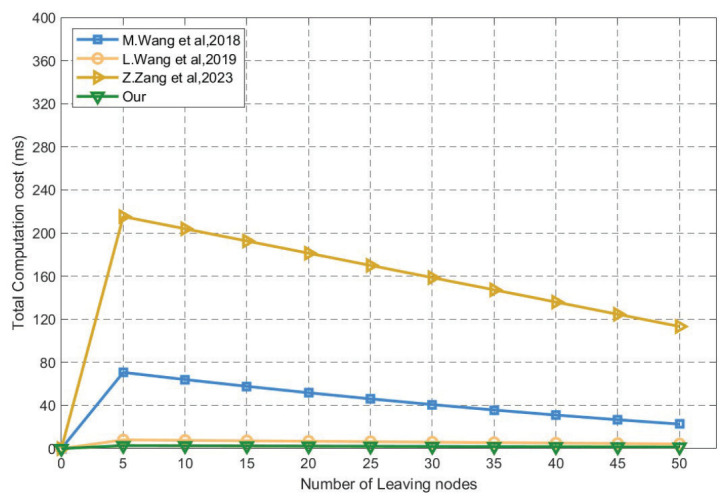
Node leaving cost comparison based on the data from Refs. [56,58,59].

We further analyse group-key-update performance. Owing to the high dynamics of group membership, we evaluate the overhead incurred when members join or leave. For a concise comparison, we set the initial group size to 35 nodes and let 5–50 nodes join in steps of 5 to measure the update cost upon member joining; we then set the initial size to 100 nodes and let 5–50 nodes leave in steps of 5 to measure the cost upon member departure. The experimental results are shown in Figure 4. It can be seen that, as the number of joining nodes increases, the computational overhead of our scheme grows more slowly than that of the other schemes. Figure 5 shows that the overhead decreases gradually as more members leave, and our cost is consistently lower. Therefore, our protocol exhibits clear advantages over the compared solutions.

### 7.2. Communication Cost Analysis

In this subsection, we compute the communication cost of our protocol and compare it with other schemes. To ensure a unified benchmark, we fix the following parameters: the elliptic-curve element size is 64 bytes; the hash output is 32 bytes; the ID and TID are 16 bytes each; the random nonces are 32 bytes; the time-stamps are 4 bytes; the ciphertext produced by symmetric encryption is 16 bytes; the PUF output is 8 bytes; the modular-exponentiation output is 128 bytes; and the Reqa request message is 8 bytes.

**Figure 6:**
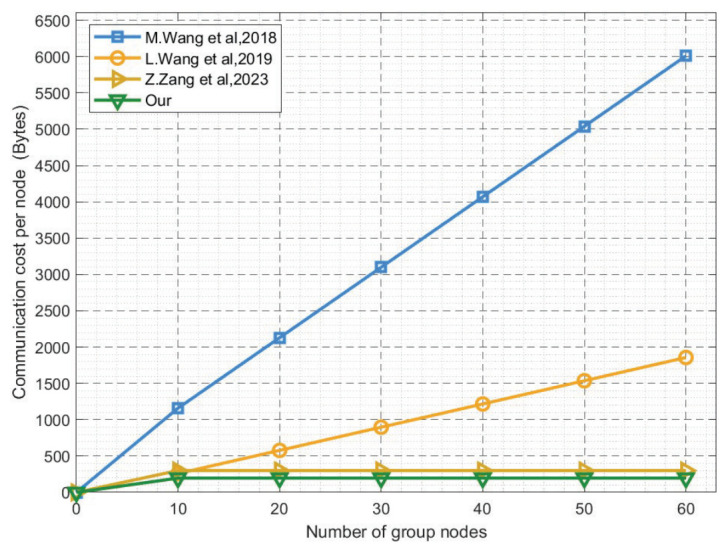
Communication cost comparison of each group node based on the data from Refs. [56,58,59].

**Figure 7:**
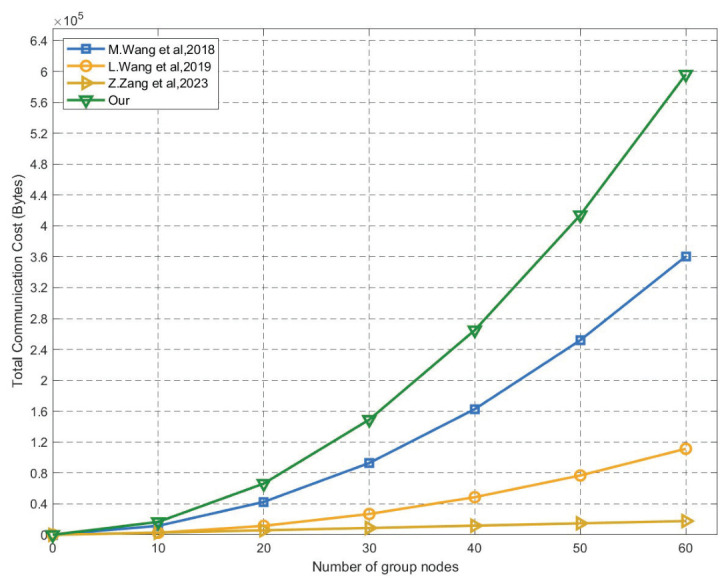
Communication cost comparison of all group nodes based on the data from Refs. [56,58,59].

Our scheme is taken as an example. During the authentication and key-agreement phase, the quantum-key cloud server first sends to each member the message $\left({\mathrm{ID}}_{q},C_{i},{\mathrm{MAC}}_{i},\;T_{a}\right)$ whose size is 16 + 16 + 32 + 4 = 68 bytes. After receiving parameter $x_{i}$, every member performs pairwise authentication and group-key agreement and needs to send $\left({\mathrm{TID}}_{i},{\mathrm{Tag}}_{i,i+1}^{0},{\mathrm{Tag}}_{i,i+1}^{1}\right)$ and $\left({\mathrm{TID}}_{i+1},{\mathrm{Tag}}_{i+1,i}^{0},{\mathrm{Tag}}_{i+1,i}^{1},{\mathrm{Tag}}_{i+1,i}^{2},t_{i+1}\right)$ whose sizes are 16 + 32 + 32 = 80 bytes and 16 + 32 + 32 + 32 + 4 = 116 bytes, respectively. Thus, the per-user communication cost is 80 + 116 = 196 bytes. In the quantum-key-request phase, the group manager sends to the quantum-key cloud server $\left({\mathrm{ID}}_{n},T_{m},{\mathrm{reqa}}_{m}\right)$ whose size is 16 + 4 + 8 = 28 bytes, while the server replies with $\left({\mathrm{MAC}}_{q},{\mathrm{ID}}_{q},T_{q},C_{i},M\right)$ whose size is 32 + 16 + 4 + 16 + 16 = 84 bytes. Finally, the group manager broadcasts the ciphertext to members, which is 16 bytes. Hence, the total group communication cost is 68n+(nln100−1)36n+196n+128bytes. The analysis of the remaining schemes is similar and omitted here. The results are listed in Table 5 and plotted in Figure 6 and Figure 7. Similarly, the communication cost comparison in Table 5 is based on the same selected schemes and should not be interpreted as a comprehensive comparison with all existing approaches. As shown in Figure 7, although the theoretical communication complexity of our scheme is $O(n^{2})$ with a larger constant factor due to the Gossip iterations, this overhead stems from a fundamentally different communication paradigm. Unlike the global broadcast mechanisms adopted in existing schemes—which suffer from severe broadcast storms, message collisions, and single-point failures as the group scale grows—our Gossip-based approach distributes communication load evenly across all nodes through localized, pairwise exchanges. This design inherently avoids network congestion caused by massive simultaneous broadcasts and ensures that a single packet loss or node failure does not compromise the entire protocol. Consequently, our scheme achieves a favorable trade-off: a moderate increase in raw communication bytes is outweighed by significant gains in scalability, fault tolerance, and robustness in large-scale, dynamic IoT deployments.

**Figure 8:**
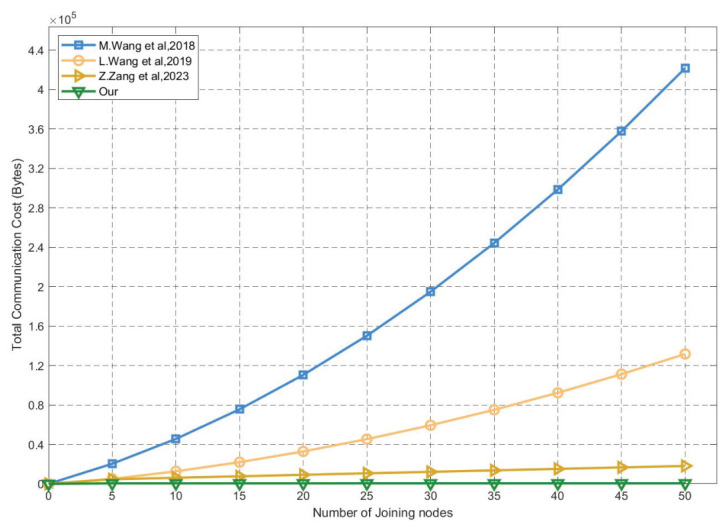
Node joining cost comparison based on the data from Refs. [56,58,59].

**Figure 9:**
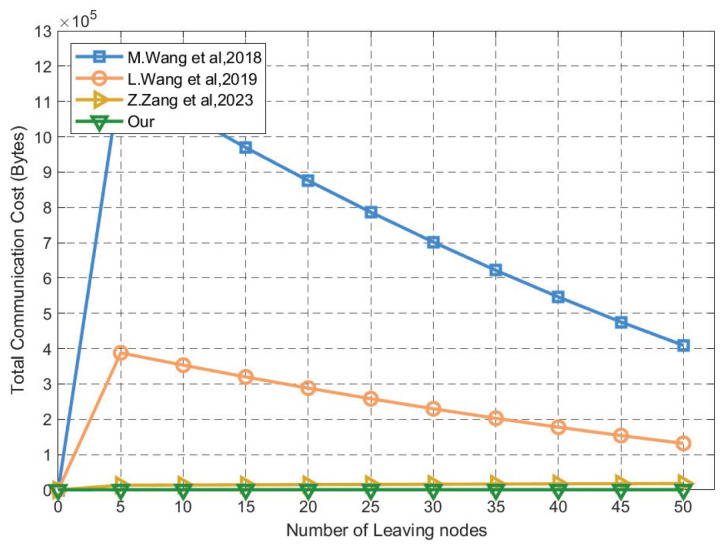
Node leaving cost comparison based on the data from Refs. [56,58,59].

We also analyze group-key-update performance. Owing to the high dynamics of group membership, we consider the overhead incurred when members join or leave. Using the same initial node numbers as in the computational-cost evaluation, we set the initial group size to 35 and let 5–50 nodes join in steps of 5 to measure the update cost upon member joining; we then set the initial size to 100 and let 5–50 nodes leave in steps of 5 to measure the cost upon member departure. The experimental results are shown in Figure 8 and Figure 9. It is evident that our scheme incurs lower communication cost than the other schemes during both member joining and leaving, demonstrating a clear advantage.

## 8. Discussion

To address the two core challenges of quantum security threats and resource constraints in the Internet of Things, this paper has proposed a lightweight anonymous group authentication scheme that integrates PUF, the Gossip algorithm, and quantum key distribution. The scheme leverages PUF to achieve hardware-level anti-forgery and anti-quantum attack capabilities, adopts Gossip-based local interaction to solve the distributed broadcast storm problem, and employs quantum keys to provide information-theoretic security protection. Meanwhile, a pseudonym mechanism is used to preserve identity privacy, and dynamic key update is adopted to accommodate dynamic group changes.

A security analysis has shown that the protocol satisfies security goals including group key security, anonymity, and forward/backward security. The PFS guarantee holds under the QKCS-trusted assumption stated in Section 4.3. The protocol also resists typical attacks such as replay attacks, man-in-the-middle attacks, and impersonation attacks. Performance evaluation demonstrates that the proposed scheme outperforms existing schemes in terms of computational cost, communication cost, and dynamic member management efficiency.

Without requiring pre-stored secret keys, the proposed scheme features lightweight computation and communication as well as strong scalability, showing promise for large-scale, highly dynamic, and resource-constrained IoT scenarios. It provides a practical and feasible technical solution for secure group communication in the IoT under the post-quantum era.

Several limitations of this study should be acknowledged. First, the performance comparison in Section 7 does not include a direct PUF-vs-PUF end-to-end comparison with existing PUF-based group key agreement schemes due to the scarcity of such works with complete complexity formulas in the literature. Second, the security analysis relies on the assumption that the QKCS remains uncompromised and that the PUF hardware exhibits stable and commutative behavior; these assumptions, while standard in the literature, may not hold in all practical deployment scenarios. Third, the experimental evaluation is conducted on a desktop-class platform rather than on actual embedded IoT hardware. Addressing these limitations—particularly through PUF-vs-PUF benchmarking, embedded hardware validation, and relaxation of the trusted QKCS assumption—constitutes our primary direction for future work. We also acknowledge that the performance comparison in Section 7 does not include a comprehensive end-to-end comparison with all PUF-based, QRNG-assisted, or Gossip-based group key schemes. The selection of baselines was limited to those with complete and reproducible complexity formulas in the literature. A broader comparison and benchmarking with more recent schemes are planned as future work.

Future work will further optimize the adaptability of the protocol in mobile scenarios and heterogeneous networks, implement a prototype on real IoT hardware platforms, and extend it to practical application scenarios with low latency and high reliability, such as the Internet of Vehicles and the industrial Internet of Things.

## Figures and Tables

**Table 1:** Notation definitions.

Symbol	Definition
${\mathrm{ID}}_{i}$	Real identity of node *i*
${\mathrm{ID}}_{q}$	Identity of the quantum-key cloud server
${\mathrm{TID}}_{i}$	The pseudonym of node *i*
${\mathrm{PUF}}_{i}\left(\cdot \right)$	Physical unclonable function of node *i*
${\mathrm{TK}}_{i}$	Temporary shared key of node *i*
H(·)	One-way hash function
${\mathrm{HMAC}}_{{\mathrm{TK}}_{i}}\left(\cdot \right)$	HMAC computed with key ${\mathrm{TK}}_{i}$
${\mathrm{SK}}_{i,i+1}$	Session key shared between node *i* and node i+1
$K_{i,i+1}$	Long-term shared key between node *i* and node i+1
$K_{i}$	Shared key between node *i* and the server
$r_{i}$	Random nonce generated by node *i*
QK	Quantum group session key
GK	Classical group session key
$E_{K}\left(M\right)/D_{K}\left(M\right)$	Encrypt/decrypt message *M* with symmetric key *K*

**Table 2:** Notations and meanings in BAN logic.

Notation	Meaning
P∣≡X	*P* believes the message *X*
P◃X	*P* sees the message *X*
P∣∼X	*P* said the message *X*
P∣⇒X	*P* has authority over *X*
#(X)	*X* is fresh
${\left\{X\right\}}_{K}$	*X* is encrypted under key *K*
$P\overset{K}{⟷}Q$	*K* is a secret key shared between *P* and *Q*
$P\overset{K}{⟺}Q$	*K* is a shared secret between *P* and *Q*

**Table 3:** Time consumption of basic operations (ms).

Symbol	Description	Time (ms)
$T_{\mathrm{puf}}$	PUF operation time	0.002956
$T_{h}$	Hash operation time	0.000872
$T_{c}$	Chebyshev chaotic map time	0.279101
$T_{\mathrm{pm}}$	Elliptic-curve point multiplication time	0.002146
$T_{s}$	Symmetric encryption/decryption time	0.002382
$T_{e}$	Modular exponentiation time	0.040927
$T_{\mathrm{hmac}}$	HMAC operation time	0.003223
$T_{\mathrm{sr}}$	Shamir secret-sharing reconstruction time	0.008609

**Table 4:** Computational cost of each scheme (ms).

Scheme	Computational Overhead	Complexity
M. Wang et al. [58]	$3nT_{e}+\left(4n+3n^{2}\right)T_{\mathrm{pm}}+nT_{h}$	$O(n^{2})$
L. Wang et al. [59]	$\left(2n+2\right)T_{e}+\left(3+n\right)T_{\mathrm{pm}}+4T_{h}$	O(n)
Z. Zang et al. [56]	$8nT_{c}+17nT_{h}+4nT_{s}+0.8nT_{\mathrm{rs}}$	O(n)
Our	$\left(2n+1\right)T_{\mathrm{puf}}+\left(n^{2}\ln100+9n\right)T_{h}+\left(3n+3\right)T_{s}+\left(2n+2\right)T_{\mathrm{hmac}}$	$O(n^{2})$

**Table 5:** Communication cost of various schemes (bytes).

Scheme	Communication Cost	Complexity
M. Wang et al. [58]	$\left(97n^{2}+189n\right)$	$O(n^{2})$
L. Wang et al. [59]	$32{\left(n-1\right)}^{2}$	$O(n^{2})$
Z. Zang et al. [56]	(300n−192)	O(n)
Our	68n+(nln100−1)·36n+196n+128	$O(n^{2})$

## Data Availability

The original contributions presented in this study are included in the article. Further inquiries can be directed to the corresponding author.
